# Tracking Chlamydia and Syphilis in the Detroit Metro Area by Molecular Analysis of Environmental Samples

**DOI:** 10.1021/acs.est.4c05869

**Published:** 2024-09-30

**Authors:** Liang Zhao, Heidy Peidro Guzman, Irene Xagoraraki

**Affiliations:** Department of Civil and Environmental Engineering, Michigan State University, 1449 Engineering Research Ct., East Lansing, Michigan 48823, United States

**Keywords:** bacterial wastewater surveillance, sexually transmitted infections (STIs), Chlamydia (*Chlamydia trachomatis*), Syphilis (*Treponema pallidum*), bacterial shedding, estimating infections

## Abstract

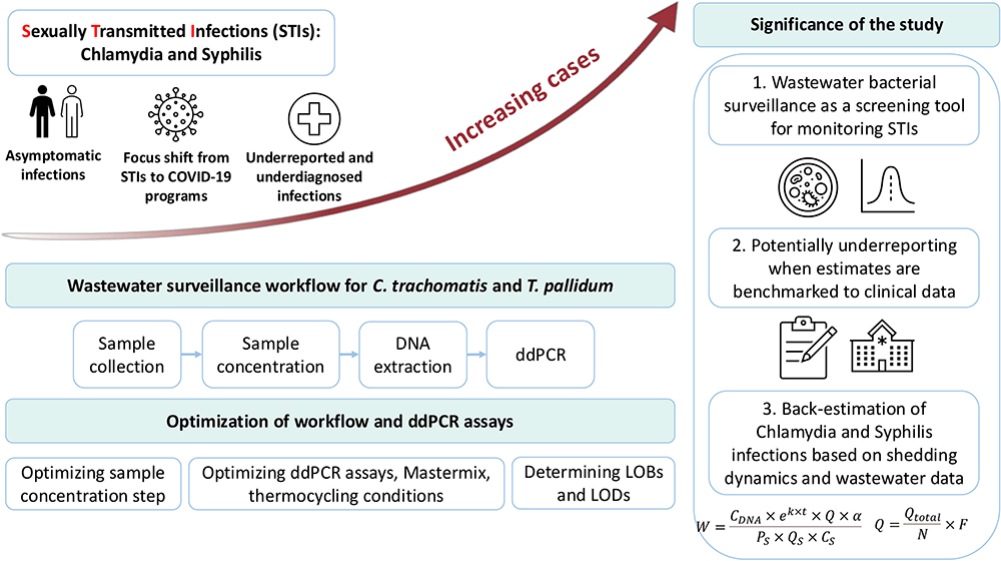

This paper describes one of the first studies applying wastewater surveillance to monitor Chlamydia and Syphilis and back-estimate infections in the community, based on bacterial shedding and wastewater surveillance data. Molecular biology laboratory methods were optimized, and a workflow was designed to implement wastewater surveillance tracking Chlamydia and Syphilis in the Detroit metro area (DMA), one of the most populous metropolitan areas in the U.S. Untreated composite wastewater samples were collected weekly from the three main interceptors that service DMA, which collect wastewater and discharge it to the Great Lakes Water Authority Water Resource Recovery Facility. Additionally, untreated wastewater was also collected from street manholes in three neighborhood sewersheds in Wayne, Macomb, and Oakland counties. Centrifugation, DNA extraction, and ddPCR methods were optimized and performed, targeting *Chlamydia trachomatis* and *Treponema pallidum*, the causative agents of Chlamydia and Syphilis, respectively. The limit of blank and limit of detection methods were determined experimentally for both targets. Both targets were detected and monitored in wastewater between December 25th, 2023, and April 22nd, 2024. The magnitudes of *C. trachomatis* and *T. pallidum* concentrations observed in neighborhood sewersheds were higher as compared to the concentrations observed in the interceptors. Infections of Chlamydia and Syphilis were back-estimated through an optimized formula based on shedding dynamics and wastewater surveillance data, which indicated potentially underreported conditions relative to publicly available clinical data.

## Abstract 2

Knowledge of wastewater surveillance methods and applications for estimating bacterial infections is limited. This paper describes one of the first wastewater surveillance efforts in monitoring Chlamydia and Syphilis and estimating community infections.

## 1 Introduction

Significant advancements in wastewater surveillance or wastewater-based epidemiology (WBE) have been achieved since the onset of the COVID-19 pandemic. Studies have shown that most respiratory, enteric, vector-borne, and bloodborne disease pathogens can be detected in wastewater and other environmental samples.^1−4^ Numerous investigations have implemented wastewater surveillance to monitor fluctuations of SARS-CoV-2 and explored its applications in comprehensive aspects.^5−16^ Recently, wastewater surveillance has also become recognized as an effective method for monitoring other viral diseases beyond COVID-19, such as norovirus, respiratory syncytial virus (RSV), and influenza viruses.^17−19^ Despite significant technological, methodological, and translational advancements in wastewater surveillance, its applications have been largely limited to monitoring viral communicable diseases encompassing adenovirus, astrovirus, enterovirus, viral hepatitis, rotavirus, poliovirus, norovirus, etc., which were summarized in a systematic review.^20^ Only two bacterial targets, *Escherichia coli* and *Salmonella*, were identified for wastewater surveillance in Kilaru et al.’s study.^20,21^ Few studies have yet explored applications of wastewater surveillance for monitoring bacterial communicable diseases. Notably, researchers monitored concentrations of *Salmonella* in municipal wastewater in Hawaii, U.S., and demonstrated positive correlations between *Salmonella* concentrations and clinical cases of Salmonellosis.^22^ Yan et al.^22^ also observed large fluctuations and outliers of *Salmonella* concentrations that presented significant challenges and uncertainties for bacterial testing in wastewater. Likewise, Matrajt et al., identified environmental surveillance methods to monitor *Salmonella* Typhi and *Salmonella* Paratyphi, which caused typhoid fever.^23^ Other researchers also conducted surveys to identify the next targets for bacterial monitoring such as *E. coli*, *Enterococci*, and *Enterobacteriaceae* in water environments.^24^ To date, only one study was identified to monitor *Chlamydia trachomatis* in wastewater on a Florida’s university campus.^25^ Philo et al. identified four major challenges facing bacterial wastewater surveillance including prioritizing new bacterial targets, establishing relationships between wastewater data and human infections, designing and developing methodologies, as well as normalizing bacterial wastewater data.^21^

The current study presents one of the first investigations using wastewater surveillance to monitor sexually transmitted infections (STIs) and provides specific solutions to some of the challenges and research gaps proposed by researchers previously regarding wastewater bacterial surveillance,^21^ including selecting new bacterial targets, relating bacterial wastewater data to human infections, and developing sampling and analytical methodologies. Recently, we developed a ranking system that prioritizes the next wastewater surveillance targets among 96 communicable diseases in the Detroit metro area.^1^ Among them, STIs, particularly Chlamydia and Syphilis were prioritized as the top fifth and seventh communicable diseases to be monitored using wastewater surveillance.^1^ Chlamydia and Syphilis are caused by Gram-negative bacteria *Chlamydia trachomatis* and *Treponema pallidum*, respectively. In 2020, the World Health Organization (WHO) estimated new Chlamydia and Syphilis infections at 128.5 million and 7.1 million, respectively.^26^ In the U.S., about 1.6 million Chlamydia cases and nearly a quarter million Syphilis cases were reported in 2022 (cdc.gov).

STIs have been rapidly increasing in the U.S., especially since the COVID-19 pandemic. The COVID-19 pandemic significantly reduced STI-related supporting staff, supplies, testing materials and medications, laboratory capacity, access to STI-related services, and surveillance activities in STI programs, which contributed to delays in STI diagnosis and treatment with concomitant increases in STI transmissions and incidences.^27,28^ Furthermore, researchers reported STI patients might have been reluctant to seek testing due to fear of COVID-19 exposure, leading to underreported STIs.^28^ Shifted focus and funds from STIs to COVID-19 programs also contributed to the underdiagnosis or under-reporting of STIs. In the State of Michigan and the Detroit metro area, Chlamydia cases have been reported in high numbers of infections annually and Syphilis infections showed significant increases between 2013 and 2023 (Figure S1). In particular, the confirmed incidences of Syphilis in Michigan and Detroit metro areas surged during the COVID-19 pandemic from 2020 to 2021.

Currently, both Chlamydia and Syphilis have reportedly been underdiagnosed and underestimated. Self-testing, opportunistic testing, and clinical testing are the commonly implemented methods to monitor Chlamydia in populations.^25^ However, these monitoring methods of Chlamydia are unable to detect the majority of infections since limited infected populations are likely to seek testing in clinical settings.^25^ Vulnerable populations are unlikely to undergo testing themselves due to a lack of education and privacy.^29^ Balfe et al. reported the following factors including the cost of testing, inconvenient STI services, long waiting times, and stigma related to STIs contributing to the difficulty of testing.^30^ Likewise, Syphilis cases are potentially underreported for similar reasons.^31^ To the best of our knowledge, there are no studies yet that investigated wastewater surveillance for *C. trachomatis* and *T. pallidum* in a major metropolitan area and performed back-estimation of Chlamydia and Syphilis infections from bacterial shedding and wastewater measurements.

In this study, untreated wastewater samples were collected weekly from three interceptors of the Great Lakes Water Authority (GLWA) in southeastern Michigan, which service the City of Detroit, Wayne, Macomb, and Oakland counties, as well as from street manholes, which service three smaller neighborhood sewersheds (EP in the Macomb county, D3 in the Wayne county, and OP in the Oakland county), between December 25th, 2023, and April 22nd, 2024. We developed a workflow encompassing sampling, centrifugation, DNA extraction, and droplet digital PCR to quantify and monitor the *C. trachomatis* and *T. pallidum* DNA concentrations in wastewater samples. We optimized assays, Mastermix, and thermocycling conditions targeting *C. trachomatis* and *T. pallidum*. We also investigated the bacterial shedding of *C. trachomatis* and *T. pallidum* and optimized a formula for estimating Chlamydia and Syphilis infections from bacterial shedding and wastewater measurements. To the best of our knowledge, this is the first wastewater surveillance study for bacterial STIs where wastewater testing was identified as a screening tool that can be followed by more targeted clinical testing in communities. The back-estimations of Chlamydia and Syphilis infections from bacterial shedding and wastewater measurements indicated likely under-reported cases, which further enhanced the significance of bacterial wastewater surveillance in monitoring STIs.

## 2 Materials and Methods

### 2.1 Positive Controls

Genomic DNA from *C. trachomatis* serovar D strain UW-3/Cx (ATCC VR-885D) was obtained from ATCC (Manassas, VA, USA). Quantitative synthetic *T. pallidum* DNA (ATCC BAA-2642SD) was obtained from ATCC (Manassas, VA, USA). Both products were immediately stored at −80 °C upon arrival. The gene copy numbers of *C. trachomatis* and *T. pallidum* standard controls were determined experimentally. We thawed both vials on ice and avoided as many freeze–thaw cycles as possible to circumvent degradation of their DNA and variation in copy numbers, by aliquoting the materials. Prior to the experiments, we gently homogenized the vials to ensure a uniform distribution of the materials and briefly centrifuged the vials.

### 2.2 Epidemiological Data

**Figure 1:**
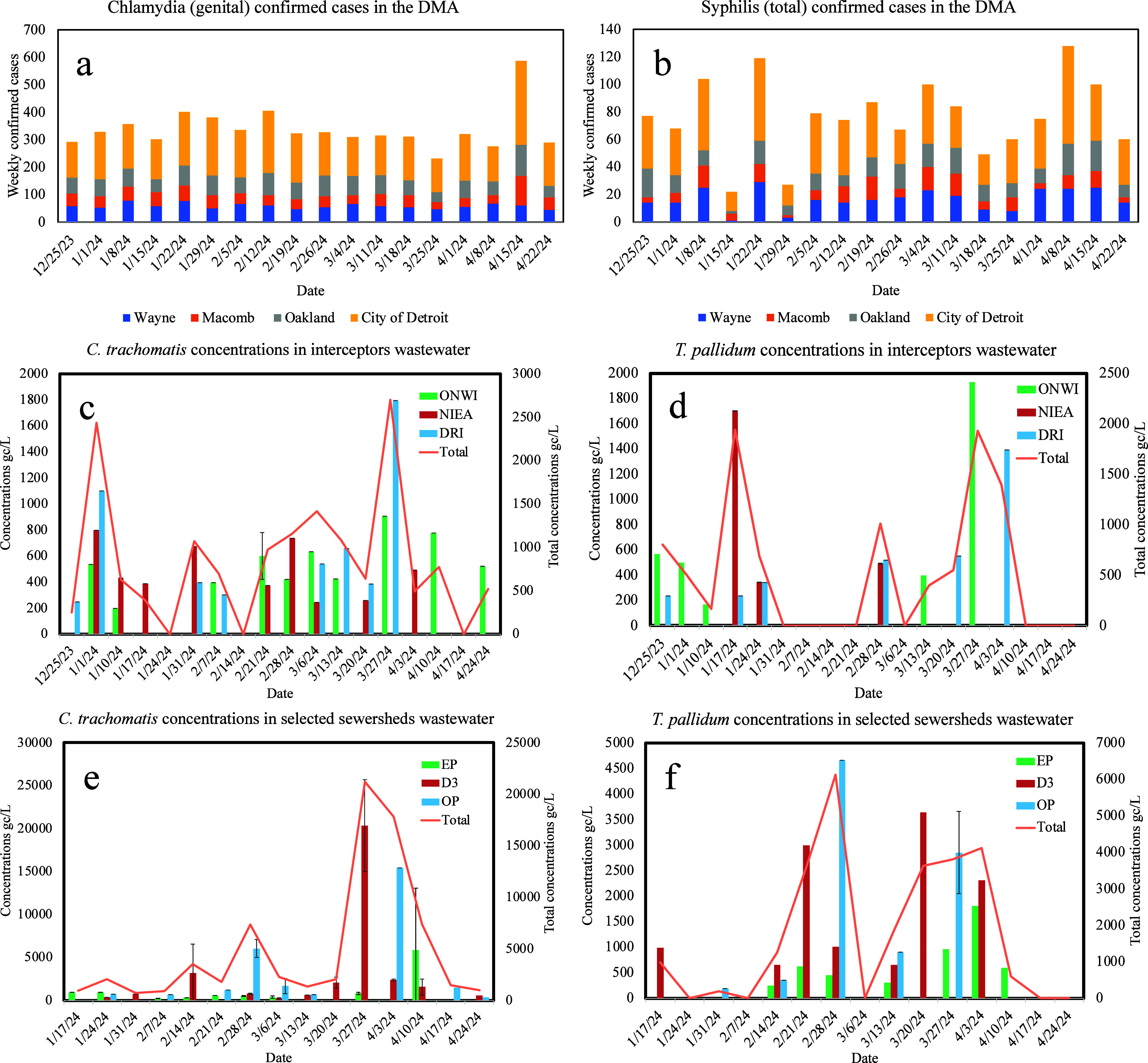
Weekly confirmed Chlamydia cases (a) and Syphilis cases (b) in the DMA, measured *C. trachomatis* concentrations in interceptors (c) and neighborhood sewershed wastewater (e) and *T. pallidum* concentrations in interceptors (d) and neighborhood sewershed wastewater (f).

Both annually (Table 1) and weekly (Figure 1a,b) reported Chlamydia and Syphilis cases for the Detroit metro area (DMA), including the City of Detroit, as well as Wayne, Macomb, and Oakland counties, were downloaded from publicly available databases of the Michigan Disease Surveillance System (MDSS) Weekly Surveillance Reports (WSR). The annually reported cases of Chlamydia and Syphilis for each jurisdiction were obtained from the annual WSR between 2013 and 2023 for MMWR week 52, which included the total cases of each disease in the year (the morbidity and mortality weekly report (MMWR) week has been established by the U.S. CDC from Sunday to Saturday and is given a sequentially increasing number from the first week in January). The weekly reported cases of Chlamydia and Syphilis encompassed a range from the MMWR week 52 of 2023 (the week of December 25th, 2023) to the MMWR week 17 of 2024 (the week of April 22nd, 2024).

**Table 1:** Annually Confirmed Cases of Chlamydia (Genital) and Syphilis (Yotal) in the Entire Detroit Metro Area (DMA) and State of Michigan (MI), City of Detroit, as well as Wayne, Macomb, and Oakland Counties, Percentage of Annual Cases of DMA within MI, between 2013 and 2023

disease	location	2013	2014	2015	2016	2017	2018	2019	2020	2021	2022	2023
Chlamydia (genital)	MI	45174	45265	48108	49417	51726	51194	49811	44547	45279	42913	42860
	DMA	21858	20658	21967	23086	23745	23289	23151	20890	20132	19882	20647
	DMA/MI percentage	48.39%	45.64%	45.66%	46.72%	45.91%	45.49%	46.48%	46.89%	44.46%	46.33%	48.17%
	Wayne	3551	3826	4196	4713	4800	4964	4817	4573	4586	4265	3850
	Macomb	2525	2494	2740	3210	3565	3581	3539	3048	3314	3063	3163
	Oakland	3530	3623	4005	4199	4220	4463	4413	3930	3130	3693	3729
	City of Detroit	12252	10715	11026	10964	11160	10281	10382	9339	9102	8861	9905
Syphilis (total)	MI	1092	1215	1116	1178	1305	1704	1893	2054	2682	2874	3215
	DMA	830	813	781	881	820	1148	1247	1334	1801	1744	1811
	DMA/MI percentage	76.01%	66.91%	69.98%	74.79%	62.84%	67.37%	65.87%	64.95%	67.15%	60.68%	56.33%
	Macomb	166	178	165	163	171	226	238	214	351	329	338
	Macomb	79	78	109	79	106	147	132	164	230	208	241
	Oakland	161	152	151	169	147	216	228	221	322	306	310
	City of Detroit	424	405	356	470	396	559	649	735	898	901	922

The Chlamydia cases reported in the WSR were denoted as “genital”. The Syphilis cases reported in the WSR included nine reportable conditions in Michigan, including congenital, early latent, late latent, latent of unknown duration, late with manifestations, primary, secondary, to be determined, and unknown duration or late syphilis. In this study, we incorporated the total cases of all reportable conditions of Syphilis, which are denoted as “Syphilis (total)”. The reported data were available for only each city or country in the Detroit metro area. Epidemiological data of Chlamydia and Syphilis for smaller jurisdictions such as zip-code areas are unavailable for the study area. Hence, only the total *C. trachomatis* and *T. pallidum* concentrations of three interceptors can be compared to the total Chlamydia (genital) and Syphilis (total) cases in the Detroit metro area.

### 2.3 Sampling Locations and Sample Collection

Sampling locations include all three main interceptors in GLWA’s WRRF and street manholes covering three neighborhood sewersheds in Wayne, Macomb, and Oakland counties (Figure S2). The WRRF consists of a semicombined system that collects and treats stormwater together with residential, industrial, and commercial waste, according to specific service areas.^9^ More details of the interceptors and their covered sewersheds are included in the Supporting Information and previous studies.^5,6,32^ Additionally, samplings at street manholes were done in three neighborhood sewersheds, including East Point (EP, ZIP code: 48021) located in Macomb County, D3 (ZIP code: 48235) located in Wayne County, and Oak Park (OP, ZIP code: 48237) located in Oakland County, with covered populations of 2400, 1300, and 2270.^8^ A total of 108 untreated 1 L 24 h composite wastewater samples were collected weekly from three interceptors and street manholes of three neighborhood sewersheds in the DMA between December 25th, 2023, and April 22nd, 2024. The samples were collected using sterilized Nalgene bottles which were then enclosed in sealed plastic bags and placed on ice. The samples were then transported to the Environmental Virology Laboratory at Michigan State University for downstream analyses within 24 h.

### 2.4 Centrifugation, DNA extraction, and ddPCR

Samples were concentrated using a modified centrifugation method, where 1 L wastewater samples were concentrated at 12,000 × *g* for 40 min.^33−36^ The concentrated samples were stored at −80 °C for downstream analyses. Bacterial DNA was extracted from the pellets using the QIAGEN DNeasy PowerLyzer PowerSoil Kit (12855–50) according to the manufacturer’s protocol with slight modifications (QIAGEN, Germantown, MD, USA). The mass of the pellets was recorded for downstream calculations. A Vortex Adapter for 24 tubes (13000-V1–24) (QIAGEN, Germantown, MD, USA) was utilized at the vortex’s maximum speed for 20 min to achieve the bead-beating process. The same QIAGEN kit was utilized for extracting *C. trachomatis* DNA from wastewater samples previously and was proven to be effective and efficient.^25^ Finally, 100 μL of bacterial DNA was extracted and stored at −80 °C for downstream analyses.

A QX200 AutoDG Droplet Digital PCR system (Bio-Rad, Hercules, CA, USA) was employed to perform ddPCR. Primers and probes targeting *ompA* and *polA* nucleotide sequences were applied to identify *C. trachomatis* and *T. pallidum*, respectively, and quantify their DNA concentrations in wastewater samples (Table S1). We implemented the same primers and probes to identify and quantify *C. trachomatis* and *T. pallidum* as used in previous studies.^25,37−41^ Optimized Mastermix reaction and thermocycling conditions are shown in Tables S2 and S3, with a total of 22 μL for each well on the 96-well plate. The target primer and probe concentrations for both assays were determined as 900 and 250 nM, respectively. Each run included positive controls for *C. trachomatis* and *T. pallidum* and negative controls using nuclease-free water.

### 2.5 Limit of Blank and Limit of Detection

Limit of blank (LOB) and limit of detection (LOD) were determined experimentally for evaluating the analytical sensitivity and validating *C. trachomatis* and *T. pallidum* assays, according to the manufacturer’s protocol (Bio-Rad, Hercules, CA, USA). Two types of samples were selected to represent blank samples in the LOB tests: nuclease-free water and autoclaved wastewater samples collected during the study period. The selection of sample types for LOB tests was predicated on the Bio-Rad protocol and previous studies.^9−11^ LOB was tested across two consecutive days for *C. trachomatis* and *T. pallidum* assays. The multiple-day testing approach examines any subtle impacts caused by tests conducted on different days.^9^ The nonparametric or rank-order method listed in the manufacturer’s protocol was chosen to further calculate LOBs since the data generated from all LOB tests demonstrated non-normal distributions. Henceforth, 96 values were ranked from the lowest to the highest and the value at the 92nd position (95% confidence) was determined as the LOB for the tested assay.^42–44^ Ultimately, LOBs for *C. trachomatis* and *T. pallidum* assays were determined as 0.07 and 0.08 gc/μL, respectively, with 95% confidence.

A sequence of dilutions for positive controls of *C. trachomatis* and *T. pallidum* ranging from 10^5^ to 10^–1^ gc/μL was performed. Subsequently, 96 replicate templates with the concentration of each dilution were tested using the assays following the nonparametric trial-error method to determine LODs as per the manufacturer’s protocol.^44^ Briefly, 96 values were ranked from the lowest to the highest, where the template concentration was determined as the LOD if less than 5% of measurements were below the predetermined LOB. Otherwise, a higher template concentration would be chosen to repeat the aforementioned tests until the final LOD was determined. Ultimately, LODs were determined as 0.125 gc/μL with 95% confidence for both *C. trachomatis* and *T. pallidum* assays.

### 2.6 Calculations of Concentrations and Back-Estimations of Infections

Concentrations of *C. trachomatis* and *T. pallidum* bacterial DNA were calculated using formula 1, based on modifications of a formula that we proposed and implemented previously.^8,9^
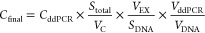
1where *C*_final_ is the concentration of bacterial DNA in wastewater samples (gc/L); *C*_ddPCR_ is the measured concentration obtained from droplet reader (gc/μL); *S*_total_ is the measured weight of final total pellets after centrifugation (gram, g); *V*_C_ is the volume of wastewater samples used for centrifugation (1 L); *V*_EX_ is the volume of extracted bacterial DNA (100 μL); *S*_DNA_ is the measured weight of pellets used for DNA extraction (g); *V*_ddPCR_ is the total volume of ddPCR final reaction for each sample (22 μL); *V*_DNA_ is the sample volume added to ddPCR Mastermix (5.5 μL).

Back-estimation of Chlamydia and Syphilis infections from *C. trachomatis* and *T. pallidum* concentrations in wastewater was performed using a modified formula 2 that was proposed in recent studies.^12,45^
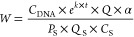
2
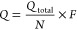
3where *W* is the number of back-estimated infections for Chlamydia and Syphilis; *C*_DNA_ is the measured DNA concentrations of *C. trachomatis* or *T. pallidum* in wastewater (gc/L); *k* is the decay rate of the bacterial DNA in wastewater (d^–1^); *t* is the wastewater in-sewer travel time (d); α is the adjustment factor involving wastewater dilution and other uncertainties discussed below; *Q* is the average wastewater generated per capita in each sewershed during the study period (L/d·person); *N* is the population captured in each sewershed; *Q*_total_ is the average wastewater flowing to each sewershed during the study period (million gallons per day, mg/d); *F* is the unit conversion factor between mg/d and L/d, which is 3.785 × 10^6^; *P*_S_ is the rate of positive detection of *C. trachomatis* or *T. pallidum* in urine from individuals of the suspected disease; *Q*_S_ is the daily volume of urine generated from an individual (mL/d·person); *C*_S_ is the shedding magnitude of bacterial DNA in urine (gc/mL).

Notably, urine-based parameters were selected for back-estimations since, unlike enteric viruses that are shed from human feces, there were limited studies reporting the shedding of both *C. trachomatis* and *T. pallidum* in human feces. Tables S8 and S9 demonstrate the positive detection rates of *C. trachomatis* and *T. pallidum* in clinical and urine samples excreted from patients with suspected diseases.^46^ We performed a global sensitivity analysis to quantify and compare the relative importance of each parameter on the final infection estimates. R package multisensi was implemented to perform sensitivity analysis on the output of the multivariate model.^47^ The details of model implementation using multisensi are included in the Supporting Information. Table S5 summarizes the decay rate constant *k* (d^–1^) for different bacteria in an aqueous environment. Among them, the range of *k* (d^–1^) for *Campylobacter* and *Salmonella* in wastewater (0.17–0.52) was selected as the approximate *k* (d^–1^) for *C. trachomatis* or *T. pallidum* in wastewater.^46^ The four bacteria share common characteristics: they are classified as Gram-negative bacteria with an outer membrane, where the *ompA* gene of *C. trachomatis* is targeted.^48^

The wastewater in-sewer travel time *t* for interceptors were obtained from GLWA (Table S6), and are 0.51, 0.94, and 0.36 days for DRI, NIEA, and ONWI interceptors, respectively. For the three smaller neighborhood sewersheds, *t* was estimated as 2.4 h or 0.1 days as per an estimation conducted in similar sewersheds of comparable size of both inhabitants and service area.^13^*P*_S_ for *C. trachomatis* was determined as 8.6%, and the average rate reported previously was between 5.3 and 14.4%.^34,49−51^*P*_S_ for *T. pallidum* was determined as 25%, and the average rate reported previously was between 12.8 and 37.1%.^40^ Additionally, due to the limited understanding of the shedding rates of *C. trachomatis* and *T. pallidum*, the full spectrum of reported urine detection rates for both bacteria was considered in back-estimating infections. *W*_min_, or the minimum number of back-estimated infections, was computed based on the maximum positive detection rates of *C. trachomatis* or *T. pallidum* in urine, which are presented in Tables S8 and S9, respectively. Likewise, *W*_max_, or the maximum number of back-estimated infections, was computed based on the minimum positive detection rates of *C. trachomatis* or *T. pallidum* in urine, which are also presented in Tables S8 and S9, respectively. *W*_ave_ was computed based on the averaged positive detection rates of *C. trachomatis* or *T. pallidum* in urine. Values marked with an asterisk (*) in Tables S8 and S9 indicate the data used for calculating positive detection rates in urine. The full range of reported detection rates of both bacteria in urine could help identify bounds on estimated cases. *Q*_S_ was estimated as 1400 mL/d·person, the average volume of urine generated by an individual, where the normal range for 24 h urine generation of an individual ranges from 800 to 2000 mL/d with an average fluid intake of about 2000 mL/d (medlineplus.gov). *C*_S_ for *C. trachomatis* was previously reported as 3.72 × 10^3^ gc/mL for urine shedding.^52^ Likewise, *C*_S_ for *T. pallidum*, was previously reported as 2.767 × 10^3^ gc/mL for urine shedding.^53^

The wastewater flows to GLWA WRRF interceptors include stormwater and residential, industrial, and commercial wastewater. The three interceptors service large sewersheds with high populations where significant dilution events can occur, including dilutions caused by industrial and commercial wastewater,^9^ stormwater,^46^ and snowmelt infiltration in March and April in Michigan.^14^ Significant decay of bacterial DNA can also occur due to long in-sewer travel time for large sewersheds.^13^ Therefore, an adjustment factor α of 100 was chosen for interceptors. The wastewater flows to manholes, covering significantly smaller sewersheds, primarily include sanitary wastewater with intermittent stormwater. Hence, an α of 10 was chosen for their adjustment.^46^ Notably, the flow data (*Q*_*total*_) for the three neighborhood sewersheds (EP, D3, and OP) were unavailable during the study period. Thus, we utilized historical flow data for D3 for the same season between January 2021 and April 2021 to approximate the flow data in the current study period. Historic flow data of EP and OP were unavailable, and we utilized historic flow data a nearby Detroit metro community (Southfield, zip-code 48076) with comparable size and population for the same season to approximate the flow data in EP and OP.^8^ It is also noteworthy that the temperature of Detroit wastewater in interceptors were estimated between 5 and 25 °C and were measured ranging from 7.6 to 21.7 °C in neighborhood sewersheds shown in Table S7.

Since the flow data for zip-code areas within the sewersheds of interceptors or selected neighborhood sewersheds were unavailable, we measured and collected PMMoV (pepper mild mottle virus) and CrAssphage (cross-assembly phage) data for the same weeks during the study period. These data were utilized to normalize *C. trachomatis* ompA and *T. pallidum* polA concentrations for comparing disparities between interceptors and selected sewersheds. The details of testing PMMoV and CrAssphage are demonstrated in the Supporting Information.

## 3 Results

### 3.1 Concentrations of *C. trachomatis* and *T. pallidum* in Wastewater of Interceptors and Neighborhood Sewersheds

*C. trachomatis* and *T. pallidum* DNA were detected in wastewater samples across three interceptors and three neighborhood sewersheds between December 25th, 2023, and April 22nd, 2024 (Figure 1). Concentrations of *C. trachomatis* DNA ranged from nondetect to 1,794 gc/L (DRI) in wastewater samples collected from interceptors, and from nondetect to 20,367 gc/L (D3) in wastewater samples collected from neighborhood sewersheds. Concentrations of *T. pallidum* DNA ranged from nondetect to 1,929 gc/L (ONWI) in wastewater samples collected from interceptors, and from nondetect to 4,664 gc/L (OP) in wastewater samples collected from neighborhood sewersheds.

For interceptors, the highest weekly concentration of *C. trachomatis* DNA was observed in the DRI interceptor (1,794 gc/L) compared to that of ONWI (908 gc/L) and NIEA (800 gc/L) interceptors. DRI interceptor primarily services the City of Detroit, where the highest weekly average cases of Chlamydia were reported as 171, compared to 59, 45, and 64, in Wayne, Macomb, and Oakland Counties. Notably, D3, which is located within the City of Detroit, demonstrated the highest weekly concentration of *C. trachomatis* DNA as 20,367 gc/L compared to that of EP (5,842 gc/L) and OP (15,416 gc/L) sewersheds. For *T. pallidum* DNA, the highest weekly concentration was observed in the ONWI interceptor (1,929 gc/L) among three interceptors (NIEA: 1,703 gc/L, DRI: 1,394 gc/L). Among the three neighborhood sewersheds, the highest weekly concentration was observed in OP (4,664 gc/L), compared to that of D3 (3,637 gc/L) and EP (1,808 gc/L).

Additionally, normalization approaches also embraced the previous findings. Both the average *C. trachomatis* and *T. pallidum* concentrations in DRI were observed higher than those of ONWI and NIEA after normalization using PMMoV and CrAssphage (Figure S6). Both *C. trachomatis* and *T. pallidum* concentrations in D3 were observed to be higher than those of EP and OP after normalization using PMMoV (Figure S7). These results indicated that higher normalized *C. trachomatis* and *T. pallidum* concentrations were observed in DRI among interceptors and D3 among selected sewersheds, which are both located within the City of Detroit.

### 3.2 Bacterial Shedding of *C. trachomatis* and *T. pallidum*

Literature review was conducted to investigate the bacterial shedding of *C. trachomatis* and *T. pallidum* in human bodily fluids. For *C. trachomatis*, the positive detection rates in human bodily fluids of people with suspected Chlamydia were reported, including an average rate of 8.6% in urine, as well as rates ranging from 8.3% to 18.4% in clinical samples such as vaginal and genital ulcer swabs.^34,49−51,54−56^ For *T. pallidum*, the positive detection rates in human bodily fluids of people with suspected Syphilis were reported including an average rate of 25% in urine, as well as rates ranging from 0.3 to 47% in clinical samples such as genital, anal ulcers and ano-rectal swabs.^38−40,54,57−59^ To our best knowledge, limited studies have reported the shedding of *C. trachomatis* and *T. pallidum* in human stool or positive detections of these bacteria in stool samples in clinical settings. The details of the reported positive rates of *C. trachomatis* and *T. pallidum* in urine and clinical samples are summarized in Tables S8 and S9, respectively.

The shedding duration of *C. trachomatis* can persist up to 10 days until the infection clears itself spontaneously.^60,61^ The incubation time of *C. trachomatis* was reported with significant variabilities from 5^62^ to 28 days.^63^ Likewise, the shedding duration of *T. pallidum* exhibited significant uncertainties and variabilities depending on the stages of syphilis, such as primary, secondary, and early latent. Researchers identified that men shed *T. pallidum* concurrently from anal and oral routes ranging from 7 to 180 days.^64^ Similarly, the incubation time of *T. pallidum* ranged from 9 to 90 days.^65^ For asymptomatic infections, it is unclear how long the incubation time would be for both STIs.

### 3.3 Back-Estimations of Chlamydia and Syphilis Infections Based on Bacterial Shedding and Wastewater Measurements

The values of the input parameters using formula 2 for back-estimating Chlamydia and Syphilis infections in interceptors and neighborhood sewersheds are shown in Table 2. Figures S9 and S10 show the sensitivity analysis indices of Chlamydia and Syphilis infection estimates, respectively. Parameters including the adjustment factor (α), average wastewater generated per capita in each sewershed (*Q*), rate of positive detection of bacteria in urine from individuals with the suspected disease (*P*_S_), shedding magnitude of bacterial DNA in urine (*C*_S_), daily volume of urine generated from an individual (*Q*_S_), and bacterial decay term (*e^kt^*), demonstrated descending importance to the infection estimates. Notably, catchment-specific characteristics including α and Q demonstrated relatively higher impacts for both Chlamydia and Syphilis infection estimates (Figures S9 and S10). The characteristics related to bacterial shedding also played a crucial role in infection estimates including Ps, Cs, Qs, which demonstrated relatively higher sensitivity indices. Further discussions on these parameters for future application and the adaptability of the model to other locations are included in the Supporting Information.

**Table 2:** Back-Estimation of Average Weekly Infections Using Formula 2

bacteria	site	*N*	*C*_DNA_	*Q*_total_	*Q*	*t*	*K*	*e^kt^*	*P*_S_	*Q*_S_	*C*_S_	α	*W*_ave_	*W*_min_	*W*_max_
*C. trachomatis*	ONWI	840600	542	231	1040.13	0.36	0.17–0.52	1.06– 1.21	ave = 8.6%, min = 5.3%, max = 14.4%	1400	3.72 × 10^3^	100	133.42–152.30	79.68–90.96	216.49–247.13
	NIEA	1482000	1482000 489	218	556.77	0.94		1.17– 1.63					71.12–99.08	42.48–59.18	115.40–160.78
	DRI	492000	679	193	1484.77	0.51		1.09– 1.3					245.35–292.62	146.53–174.76	398.12–474.82
	EP	2400	1176	0.19	299.65	0.1		1.02– 1.05				10	8.03–8.26	4.79–4.93	13.02–13.40
	D3	1300	2997	0.8	2329.23								158.98–163.65	94.94–97.74	257.96–265.55
	OP	2270	3148	0.19	316.81								22.71–23.38	13.56–13.96	36.85–37.94
*T. pallidum*	ONWI	840600	713	231	1040.13	0.36		1.06– 1.21	ave = 25%, min = 12.8%, max = 37.1%		2.767 × 10^3^	100	81.17–92.66	54.70–62.44	158.54–180.97
	NIEA	1482000	847	218	556.77	0.94		1.17– 1.63					56.97–79.37	38.39–53.49	111.28–155.02
	DRI	492000	546	193	1484.77	0.51		1.09– 1.3					91.24–108.82	61.48–73.33	178.21–212.54
	EP	2400	713	0.19	299.65	0.1		1.02– 1.05				10	2.25–2.32	1.52–1.56	4.39–4.52
	D3	1300	1749	0.8	2329.23								42.91–44.17	28.91–29.76	83.80–86.27
	OP	2270	1793	0.19	316.81								5.98–6.16	4.03–4.15	11.69–12.03

For interceptors, it is noteworthy that the highest estimated infections of both Chlamydia and Syphilis were observed in DRI, despite the fact that DRI services the smallest number of inhabitants, as compared to the other two interceptors. For neighborhood sewersheds, the highest estimated infections of both Chlamydia and Syphilis were observed in D3, despite the fact that D3 services the lowest number of inhabitants compared to the other tested sewersheds. Notably, DRI services the majority of the City of Detroit and the sewershed covered by D3 is also located within the City of Detroit. These estimated Chlamydia and Syphilis infections based on wastewater measurements, agree with the trends of confirmed cases of Chlamydia and Syphilis in DMA, where the highest weekly confirmed cases were observed in the City of Detroit (Table 1).

Additionally, the comparisons between the ranges of estimated average weekly Chlamydia and Syphilis infections for interceptors and weekly confirmed cases for their approximate regions are demonstrated in Table 3. Notably, the weekly confirmed cases for both Chlamydia and Syphilis for all approximate regions were potentially underreported compared to the back-estimated weekly infections for the interceptors, with only one exception for the Chlamydia estimation at the NIEA interceptor. The ratio between ranges of estimated weekly infections and weekly confirmed cases for approximate regions was computed (Table 3). *W*_ave_/*C*_ave_ ratios for both Chlamydia and Syphilis are above 1 except for the NIEA interceptor. For Syphilis, values of *W*_ave_/*C*_ave_ were consistent at approximately around 3 for NIEA, DRI, and total interceptors. Overall, for Syphilis, higher values of the three ratios (*W*_ave_/*C*_ave_, *W*_min_/*C*_ave_, and *W*_max_/*C*_ave_) were observed (all above 1) as compared with those for Chlamydia.

**Table 3:** Comparison between Back-Estimated Weekly Cases in the Sewersheds Serviced by Interceptors and Average Weekly Confirmed Cases in the Approximate Regions in the Detroit Metro Area

disease	interceptors	estimated average weekly cases (W_ave_)	estimated minimum weekly cases (*W*_min_)	estimated maximum weekly cases (*W*_max_)	confirmed average weekly cases (*C*_ave_) for approximate regions	ratio *W*_ave_/*C*_ave_	ratio *W*_min_/*C*_ave_	ratio *W*_max_/*C*_ave_
Chlamydia	ONWI	133.42–152.30	79.68–90.96	216.49–247.13	Wayne county: 58.72	2.27–2.59	1.36–1.55	3.69–4.21
	NIEA	71.12–99.08	42.48–59.18	115.40–160.78	Oakland and Macomb counties: 109.06	0.65–0.91	0.39–0.54	1.06–1.47
	DRI	245.35–292.62	146.53–174.76	398.12–474.82	City of Detroit: 170.9	1.44–1.71	0.86–1.02	2.33–2.78
	total	449.89–544.00	268.69–324.89	730.01–882.72	total: 338.7	1.33–1.61	0.79–0.96	2.16–2.61
Syphilis	ONWI	81.17–92.66	54.70–62.44	158.54–180.97	Wayne county: 16.44	4.94–5.64	3.33–3.80	9.64–11.01
	NIEA	56.97–79.37	38.39–53.49	111.28–155.02	Oakland and Macomb counties: 23	2.48–3.45	1.67–2.33	4.84–6.74
	DRI	91.24–108.82	61.48–73.33	178.21–212.54	City of Detroit: 37.22	2.45–2.92	1.65–1.97	4.79–5.71
	total	229.39–280.85	154.57–189.25	448.02–548.54	total: 76.67	2.99–3.66	2.02–2.47	5.84–7.15

## 4 Discussion

### 4.1 Wastewater Surveillance as a Screening Tool for Chlamydia and Syphilis

This study demonstrates the utility of wastewater surveillance on monitoring STIs, particularly Chlamydia and Syphilis in wastewater in a large urban center as well as in small neighborhood sewersheds. Recent studies indicated that factors such as the lack of willingness of patients to be tested, asymptomatic STIs, and shift of focus from STIs to COVID-19 programs contributed to uncertainties in the accuracy of clinically reported cases of Chlamydia and Syphilis.^27,28^ Particularly, due to the high infectivity and rapid transmission of STIs in densely populous areas such as Detroit, universal screening, regardless of asymptomatic or symptomatic conditions, in clinical settings is practically impossible. Thus, wastewater surveillance for Chlamydia and Syphilis may highlight fluctuations in STIs in communities, complementing clinically reported cases.

Notably, higher DNA concentrations of both *C. trachomatis* and *T. pallidum* were observed in the three smaller neighborhood sewersheds as compared to the three interceptors that cover the entire DMA, which can be likely attributed to different scales of dilution, especially since the DMA collection network is a semicombined sewer overflow system. Also, researchers indicated that longer in-sewer travel time in larger sewersheds led to greater variabilities and degradation of pathogenic concentrations in wastewater with potentially 50% or more signals degrading.^13^ In particular, the size and in-sewer travel time of interceptors (ONWI, NIEA, and DRI) are significantly larger than those of the smaller neighborhood sewersheds (EP, D3, and OP).

### 4.2 Potential Underreporting of Chlamydia and Syphilis Incidences when Wastewater Surveillance Estimates Are Benchmarked against Clinical Data

This study demonstrates the possibilities of back-estimating Chlamydia and Syphilis infections based on wastewater data and bacterial shedding using a modified formula.^45^ Comparative analyses between ONWI-interceptor estimated infections and clinical cases reported in Wayne county, NIEA-interceptor estimated infections and clinical cases in combined Oakland and Macomb counties, and DRI-interceptor estimated infections and City of Detroit clinical cases were carried out, and results are shown in Table 3. For Chlamydia infection estimates, the confirmed weekly cases were likely underreported for some sewersheds serviced by interceptors, including OWNI-sewershed. For instance, the total estimated average infections (ranging from 449.9 to 544 for Chlamydia and from 229.4 to 280.9 for Syphilis) indicated that the total confirmed weekly cases (338.7 and 76.67 for Chlamydia and Syphilis, respectively) for the DMA are likely underreported in the Michigan Disease Surveillance System (MDSS) weekly surveillance reports (WSR). The higher infection estimates based on wastewater surveillance, potentially imply that the weekly confirmed case data represent only a portion of the total infections in communities. The ratio between Syphilis infection estimates and corresponding clinical data ranged from 1.65 to 11.01, which also demonstrates higher infection estimates compared to reported clinical data (Table 3). These results potentially embraced previous findings where researchers indicated that Chlamydia and Syphilis have been underdiagnosed and underreported due to asymptomatic infections and inadequate participation by infected individuals.^25,66^ STIs such as Chlamydia and Syphilis remain underdiagnosed and untreated in communities and have the potential to become widespread without a comprehensive screening method for both symptomatic and asymptomatic infections. To address the issues above, the workflow of bacterial wastewater surveillance monitoring both Chlamydia and Syphilis proposed in this study can be an ideal screening method.

### 4.3 Concentrations of *C. trachomatis* and *T. pallidum* Might Relate to Socioeconomic Demographics

Among the three neighborhood sewersheds EP, D3, and OP, it was observed that D3 presented significantly higher estimated infections of both Chlamydia (158.98–163.65) and Syphilis (42.91–44.17), despite having the lowest population. Particularly, D3 exhibited distinctive demographic characteristics among the three neighborhood sewersheds. These demographic characteristics of the D3 sewershed include the highest poverty percentage, and the highest population density, in contrast to OP and EP sewersheds (Table S11). Likewise, researchers identified that a low socioeconomic status generally relates to limited healthcare access. STI rates generally relate to social determinants.^67,68^ Notably, the highest ranges of estimated infections for both Chlamydia and Syphilis were observed in D3 and DRI (Table 2), both located within the City of Detroit. Nevertheless, the clear connection between higher infection rates of both diseases and socioeconomic demographics requires further investigation.

### 4.4 Limitations and Future Directions

This study demonstrates one of the first workflows for bacterial wastewater surveillance and presents the detection of *C. trachomatis* and *T. pallidum* in wastewater as a screening method. We adopted a centrifugation method to concentrate and isolate bacteria in wastewater. However, the limitation of this method is the potential losses of bacteria in the supernatant.^69^ We initially implemented a membrane filtration method using 0.2 μm filters, but results were not presented due to low recoveries. We initially tested *C. trachomatis* and *T. pallidum* in supernatant wastewater, but their recoveries were low. Researchers identified that the direct centrifugation method demonstrated a higher recovery rate when compared to filtration coupled with centrifugation.^36^ Bacterial cells can often be isolated using centrifugation with a speed of more than 8000 × *g*;^69^ previous studies concentrating bacteria from environmental and clinical samples using centrifugation were summarized in Table S4.

Some parameters that were implemented in the back-estimation model still need further investigation. For instance, there have been very limited studies reporting the parameter of shedding magnitude Cs for urine, which was identified as a limitation of the current study. Consequently, future research is needed to investigate the magnitude of Cs shedding in urine, feces, and other human bodily fluids of suspected infections. Besides, for selected sewersheds, using the measured flow data (*Q*) instead of the historical flow data would potentially reduce the uncertainty of estimates.

Albeit the wide applications of PMMoV and CrAssphage as biomarkers for the normalization of wastewater pathogenic concentrations, the relative recovery of their signal may differ from the recovery of gene concentrations of *C. trachomatis* and *T. pallidum*. In addition, differences in genomes (RNA versus DNA) may also affect the normalization outcomes. Therefore, in addition to these human fecal indicators, investigations on other closely related biomarkers are needed. Future research is also needed to address gaps in types of process, recovery, and inhibition controls when wastewater surveillance is expanded to monitoring bacterial targets.^21^

It is critical to indicate that neither infection estimates based on wastewater surveillance nor clinically reported cases may depict actual infections in communities. Wastewater surveillance estimates could be beneficial when clinical testing capacity is limited, asymptomatic infections are dominating, or the willingness of individuals to participate in clinical testing is low. However, wastewater surveillance estimates may not provide sufficient data for the unsewered areas. Other sources of data can also be used to compare with wastewater surveillance estimates and clinical data, including Google trends, digital epidemiological data, mobility data, reports published by health departments or the WHO, census statistics, data obtained from scientific studies, and data in news and media.^70^ Data from these aforementioned databases are useful sources for tracking disease infections in communities.

Several questions still remain to be further investigated, including whether bacterial cells of *C. trachomatis* and *T. pallidum* may grow or decay in wastewater and how the temperature affects the decay of bacterial targets in wastewater. There is little published research investigating the growth and decay of *C. trachomatis* and *T. pallidum* in wastewater. Both *C. trachomatis* and *T. pallidum* are classified as Gram-negative bacteria that replicate only within the host cells but not in the environment.^71−73^*C. trachomatis* cannot replicate outside the human host cells. Likewise, *T. pallidum* is an obligate human pathogen; animal reservoirs for *T. pallidum* have not been reported yet, which enhances its dependence on human host cells.^73,74^ Both *C. trachomatis* and *T. pallidum* rely entirely on nutrients from the human host cell.^75,76^ However, to date, there is limited research investigating the bacterial growth of *C. trachomatis* and *T. pallidum* in wastewater.

## 5 Environmental Implications

This study fills multiple important knowledge gaps in the field of wastewater surveillance. First, this study demonstrates one of the first wastewater surveillance applications in monitoring widespread STIs, particularly Chlamydia and Syphilis, in a large urban area, as well as neighborhood sewersheds. This study established a workflow of implementing bacterial wastewater surveillance, where molecular biology laboratory methods were optimized to detect and quantify *C. trachomatis* and *T. pallidum* in wastewater. The results highlight the utility of wastewater surveillance as a screening tool to complement the clinically reported cases of bacterial diseases. Second, the results of different concentrations of *C. trachomatis* and *T. pallidum* in wastewater in different sewersheds, demonstrate disparities in corresponding contributing populations. Third, Chlamydia and Syphilis infections were back-estimated using a modified formula based on investigations on shedding dynamics of *C. trachomatis* and *T. pallidum* in environmental and clinical samples, revealing potentially under-reported cases of both diseases in the Detroit metro area.

## Supporting Information Available

Details on wastewater interceptors, a map describing sampling sewersheds and locations, as well as demographic characteristics of the selected neighborhood sewersheds; laboratory method details, such as LOB, LOD, primers and probes, Mastermix, and thermocycling conditions; information on uncertainty analysis, sensitivity analysis, and parameters of back-estimation model; approaches to compare concentrations between interceptors and selected neighborhood sewersheds, including graphs of normalized data; experimental details and results of PMMoV and CrAssphage; temperature and pH of the wastewater samples collected from the selected neighborhood sewersheds; literature review information on detection rates of *C. trachomatis* and *T. pallidum* in clinical samples (PDF)
