# Supplementary material for: Tracking Chlamydia and Syphilis in the Detroit Metro Area by Molecular Analysis of Environmental Samples
Source: Environ Sci Technol. 2024 Sep 30;58(40):17606–16. doi: 10.1021/acs.est.4c05869 (PMC11465648; doi:10.1021/acs.est.4c05869)
Supplement: Supplementary file 1 — es4c05869_si_001.pdf [file es4c05869_si_001.pdf]

Supporting Information

Tracking Chlamydia and Syphilis in the Detroit metro area by molecular analysis of environmental samples

Authors

Liang Zhao, Heidy Peidro Guzman, Irene Xagoraraki*

Affiliations

Department of Civil and Environmental Engineering, Michigan State University, 1449 Engineering Research Ct., East Lansing, MI 48823, USA

*Corresponding author: Irene Xagoraraki (xagorara@msu.edu)

Number of pages: 35

Number of Tables: 12

Number of Figures: 10

Additional details on GLWA WRRF interceptors. The three main GLWA WRRF interceptors service an area of more than 946 square miles covering the City of Detroit and the three most populous counties in Michigan, including Wayne, Macomb and Oakland counties, with a total population of approximately three million. Three interceptors are: the Oakwood-Northwest-Wayne County Interceptor (ONWI), the North Interceptor-East Arm (NIEA), and the Detroit River Interceptor (DRI), which service approximately 840,600, 1,482,000, and 492,000 inhabitants, respectively, based on 2020 census data. The interceptors convey large volumes of wastewater to the GLWA wastewater treatment facilities.

Additional details on primers and probes. Previous studies utilized qPCR in testing *C. trachomatis* and reported average efficiencies of 87.4% in wastewater samples (1) and 88.9% in clinical samples (2). Likewise, researchers utilized qPCR or real-time PCR in testing *T. pallidum* where they observed high sensitivities and specificities of 89% to 100% in clinical samples (3–6).

Additional details on LOB and LOD. The Bio-Rad protocol was developed predicated on the Clinical and Laboratory Standards Institute (CLSI) protocol "EP17 Evaluation of Detection Capability for Clinical Laboratory Measurement Procedures" (7). The CLSI EP17 document suggested at least 60 replicate templates to determine LOBs (7). Thus, 96 replicate templates were conducted for both types of samples to determine LOBs for *C. trachomatis* and *T. pallidum* assays, respectively.

Additional details of recovery. Bacteriophage Phi6 prepared in the PCR-grade water and Buffer AVL were used as a proxy agent to estimate losses during concentration and RNA extraction. The recoveries obtained were from 10.37 % to 58.96 %, with a mean recovery of 24.91 % (15).

Target	Name	Sequence	Reference
Phi6	Φ6Tfor	5'-TGGCGGCGGTCAAGAGC-3'	(42)
	Φ6Trev	5'-GGATGATTCTCCAGAAGCTGCTG-3'	
	Φ6Tprobe	5'- FAM-CGGTCGTCGCAGGTCTGACACTCGC-BHQ1-3'	

Additional details on the uncertainty analysis. We adopted a commonly used statistical method of propagation of uncertainty to estimate the effect of each variable's uncertainty on the uncertainty of the estimated infection (W) using for formula (2) for interceptors. We did not include the neighborhood selected sewersheds (EP, D3, OP) in the analysis since we did not have the required flow data and wastewater travel time records. The uncertainty analysis for interceptors can sufficiently demonstrate the disparities of uncertainties of each parameter in formula (2) and their impacts on estimated infections. In formula (2), we first identified the uncertainty of each independent variable, they are: σ (C_{DNA}), σ (k), σ (t), σ (Q), σ (α), σ (Ps), σ (Qs), and σ (Cs). When we focused on the targeted variable for calculating its uncertainty for the estimated infections, we would neglect the uncertainties of remaining variables. We computed the partial derivatives of W with respect to each variable. The following equation is an example of computing the partial derivative of W with respect to the measured concentration C_{DNA}.

$$\frac{\partial W}{\partial C(DNA)} = \frac{e^{kt}Q\alpha}{PsQsCs}$$

$$\frac{\partial W}{\partial k}=\frac{C_{DNA}te^{kt}Q\alpha}{PsQsCs}$$
$$\frac{\partial W}{\partial t}=\frac{C_{DNA}ke^{kt}Q\alpha}{PsQsCs}$$
$$\frac{\partial W}{\partial Q}=\frac{C_{DNA}e^{kt}\alpha}{PsQsCs}$$
$$\frac{\partial W}{\partial \alpha}=\frac{C_{DNA}e^{kt}Q}{PsQsCs}$$
$$\frac{\partial W}{\partial Ps}=-\frac{C_{DNA}e^{kt}Q\alpha}{(Ps\hat{}2)QsCs}$$
$$\frac{\partial W}{\partial Qs}=-\frac{C_{DNA}e^{kt}Q\alpha}{(Qs\hat{}2)PsCs}$$
$$\frac{\partial W}{\partial Cs}=-\frac{C_{DNA}e^{kt}Q\alpha}{(Cs\hat{}2)QsPs}$$

Subsequently, the uncertainty of each variable is determined as described below. σ (C_{DNA}) is determined as the LOD for both targets (0.125). σ (k) is determined as the standard deviation (0.25) of identified k values used in Table 2. σ (t) is determined as the standard deviation of tranportation time of wastewater for each interceptor: ONWI (0.27), NIEA (0.53), and DRI (0.43). σ (Q) is determined as the standard deviation of flow data for each interceptor: ONWI (88.39), NIEA (114.47), and DRI (80.58). Qs is regraded as a constant and its undertainty σ (Qs) is zero. There is a lack of research on Cs in the current literature, and we have identified it as a future research need. Finally, through the computation of derivatives of W with respect to each parameter, the following two tables summarized uncertainties of each parameter based on values shown in Table 2. It can be seen that the uncertainties of measured concentrations are negligible compared to those of other variables since the precision of measured concentrations relied on LOD of experiments. Other variables including k, t, and Q are computed via standard deviation according to a series of collected data and they presented similar levels of uncertainties. Values of Ps were adopted from literature studies and exhibited significant variations, therefore leading to the highest contribution to uncertainties of the infection estimates.

C. trachomatis σ (W)	ONWI		NIEA		DRI	
σ (C_{DNA})	0.031	0.035	0.018	0.025	0.045	0.035
σ (K)	12.01	13.71	16.71	23.28	31.28	37.31
σ (t)	12.43	14.19	13.00	8.55	36.40	43.41
σ (Q)	11.34	12.94	14.62	20.37	13.32	15.88
σ P_S	4803.40	5483.13	2560.51	3567.21	8833.06	10534.85

T. pallidum σ (W)	ONWI		NIEA		DRI	
σ (C_{DNA})	0.014	0.016	0.008	0.012	0.021	0.025
σ (K)	7.31	8.34	13.39	18.65	11.63	13.87
σ (t)	7.56	8.63	10.42	14.51	13.54	16.14
σ (Q)	6.90	7.87	11.71	16.32	4.95	5.91
σ P_S	5579.01	6368.49	3915.79	5455.33	6271.23	7479.44

Additional detailes on the sensitivity analysis. We adopted the global sensitivity analysis using the R package multisensi to estimate the sensitivity and relative importance of each parameter in

the model on the final infection estimates (8). For sensitivity analysis, data ranges of parameters were described in the manuscript. Briefly, the decay rate term e^{kt} was denoted as “E” and is ranging from 1 to 2 based on Table 2. The data range of Q is determined from 300 to 2500. The adjustment factor was denoted as “a”. For *C. trachomatis*, Ps is ranging from 0.05 and 2. For *T. pallidum*, Ps is ranging from 0.128 to 0.371. Qs is ranging from 800 to 2000 as per descriptions in the manuscript. As per the values demonstrated in Table 2 and the details described in the manuscript of sensitivity model inputs, dynamics of the sensitivity indices for all parameters were demonstrated in the Figures S9 and S10 for Chlamydia and Syphilis infection estimates, respectively.

Additional discussions on the parameters of the back-estimation model. The adjustment factor α was respectively determined for three Detroit’s GLWA interceptors as well as the three selected sewersheds in terms of significant differences in dilution effects and sanitary percentage of wastewater. For the three interceptors, the sanitary percentage (SF) of wastewater was observed as low as 8% for the ONWI interceptor; as low as 18% SF of wastewater for the NIEA interceptor; as low as 9% SF of wastewater for the DRI interceptor, during the study period. GLWA WRRF’s interceptors consist of semi-combined sewershed systems, which receive sanitary wastewater, industry wastewater, stormwater, as well as other sources of commercial wastewater covered in an extensive area of the Metro Detroit region encompassing three largest Michigan counties (Wayne, Macomb, and Oakland) as well as City of Detroit. Therefore, expected significant dilution effects can occur. In contrast, untreated wastewater samples collected from street manholes of the three selected sewersheds cover smaller communities with 100% of sanitary wastewater, where the sampling location is also closer to the shedding sources. There were no input of industry wastewater, commercial wastewater or other sources of wastewater into the manholes. Therefore, the dilution effects of the three selected sewersheds are expected to have much less impact on the back-estimation model. The parameter α can be selected based on parameters including sewershed size, dilution effects, sanitary percentage of wastewater, when the back-estimation model is adapted to another given location. Nevertheless, more research is needed to investigate the adjustment parameter α, such as hydrological models, for adaptability of the model beyond the given locality.

Approaches to compare concentrations between interceptors and selected sewersheds. PMMoV presents in human feces due to consumption of pepper products and it is stable in wastewater with little seasonal variation (9). CrAssphage is a bacteriophage that pervasively infects the human gut commensal bacteria and is excreted into wastewater (10). Both PMMoV and crAssphage are proven to be the most consistent biomarkers and human fecal indicators in wastewater, and were implemented in recent studies to normalize wastewater viral (i.e., SARS-CoV-2, norovirus GI/GII, astrovirus), bacterial (i.e., *Campylobacter jejuni*, *Clostridioides difficile*, *Salmonella* spp., *Yersinia enterocolitica*), fungal (i.e., *Blastocystis* spp.), and protozoan (i.e., *Balantidium coli*) concentrations to mitigate the influence of systematical variations due to routine WWTPs operations, reduce background noise, account for dilution effects and enhance comparability among sites (10–13). Both PMMoV and crAssphage were highly associated with large solids collected by centrifugation, which is the concentration method adopted for isolation of *C. trachomatis* and *T. pallidum* in the current study (14). Therefore, both targets were selected for normalizing *C. trachomatis ompA* and *T. pallidum polA* concentrations for comparing disparities between interceptors and selected sewersheds.

Additional details of testing PMMoV and crAssphage in wastewater. ddPCR was performed on a QX200 AutoDG Droplet Digital PCR system (Bio-Rad, Hercules, CA, USA), using the One-step Advanced Kit for Probes (Bio-Rad, Hercules, CA, USA) and the GT-Digital PMMoV CrAssphage Assay Kit (catalog number: 100608-1.1). For the Mastermix, 1.1 μL GT-Assay solution (20X) and 6.6 μL RNase free water were included. 5.5 μL targeted template was analyzed for each sample. The thermocycling conditions are presented as follows: i) 25°C for 3 min; ii) 50°C for 60 min; iii) 95°C for 10 min; iv) 39 Cycles* of: (1) 94°C for 30 sec*, (2) 55°C for 1 min*; v) 98°C for 10 min; vi) 4°C for ∞#. Notes: NO hot start, 40 μL reaction, *Slow ramp speed of 2°C/second; #Hold: for at least 30 mins before transferring to plate reader. The Limit of Blank (LOB) was determined for PMMoV as 0.09 gc/μL, and crAssphage as 0 gc/μL, as per experiments designed previously (15,16). The Limit of Detection (LOD) for both targets were determined as 0.2 gc/μL with more than 95% confidence (15,16).

Additional details on performing alternative bacterial concentration method. In addition to the concentration method we described in the manuscript, we initially adopted a filtration method. We briefly describe the method as follows: Filtration was done using 150 mL ThermoFisher Scientific Nalgene Sterile Analytical Filter Units (130-4020) and each sample was poured into its respective filter unit while it was attached to a vacuum according to the EPA standard protocol method 1603 (1,17). The filters were removed from the unit and stored at -80°C until DNA extraction. Subsequently, we did a comparative analysis of analyzing the targeted pathogen concentrations (for both Chlamydia and Syphilis) in pellets and supernatant wastewater after centrifugation, as well as filters of different sizes after filtration, for the same samples. Specifically, we analyzed untreated 3L 24-hour composite wastewater samples collected using the aforementioned sampling method from all three interceptors for three continuous weeks in December 2023, including the weeks of 12/11/23, 12/18/23, 12/25/23. We first homogenized each sample by mixing. Then we took 100 mL of the sample to pass through 0.2 μm filter. It is important to indicate that all untreated wastewater samples exhibited high amounts of solids and clogged filters completely for any amount of wastewater above 100 mL even with subsequently added pre-filtration step. The filtered-samples were marked as sample type A. From the same 3L sample, we took 1L sample to perform centrifugation at 12000 xg for 40 minutes, following separation of pellets and supernatant wastewater. For supernatant wastewater, we passed 300 mL of supernatant through 0.2 μm filter and the newly-filtered-samples were marked as sample type B. The pellet sample was marked as sample type C. We tested all three types of samples for all three interceptors for three continuous weeks for targeted pathogens. Consequently, higher concentrations and much higher number of positive samples were detected in wastewater type C samples. We finally proceeded with centrifugation method for concentration since the results of this method is consistent and the higher concentrations/higher number of positive samples can be detected using this approach. Additionally, centrifugation was commonly used in concentrating environmental and clinical samples for the detection of bacterial targets, including *C. trachomatis*, *Salmonella* Typhi, *E. coli*, *Legionella pneumophila*, *Enterococcus faecalis*, and *Leptospira*, which were summarized in Table S1.

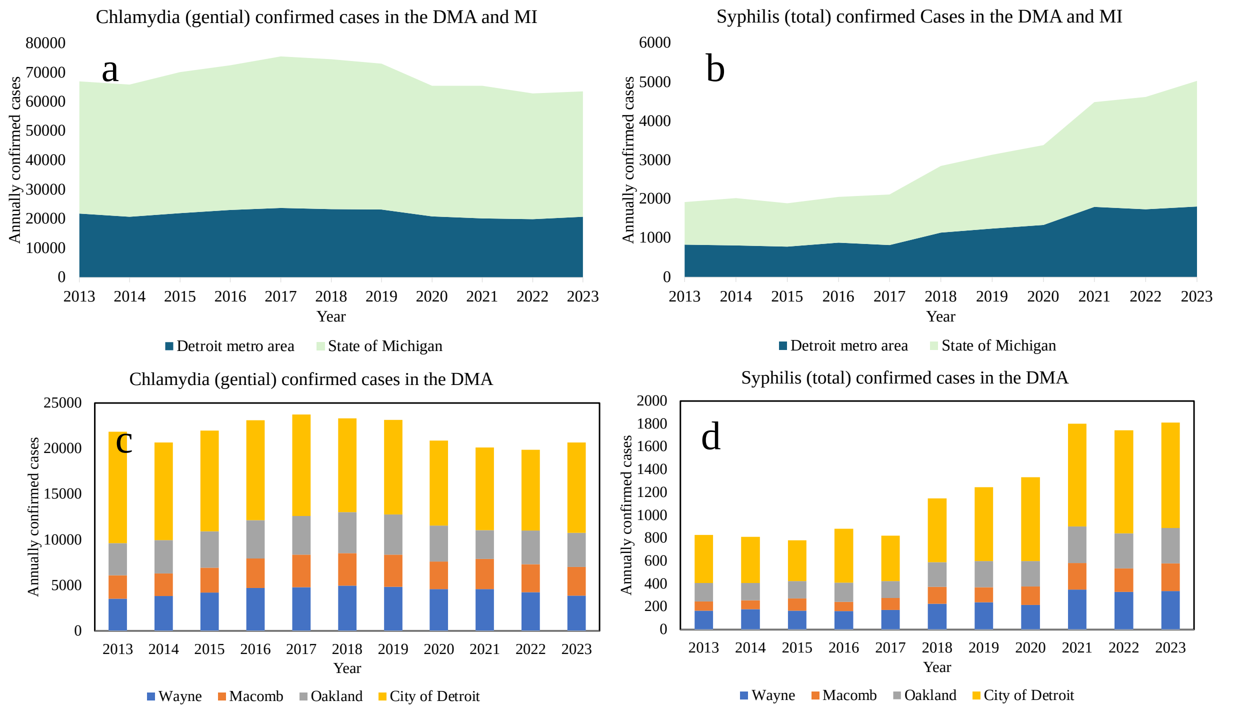

Figure S1. Annually confirmed cases of Chlamydia (genital) and Syphilis (total) in the entire Detroit metro area and State of Michigan (a, b), City of Detroit, as well as Wayne, Macomb, and Oakland counties (c, d) between 2013 and 2023

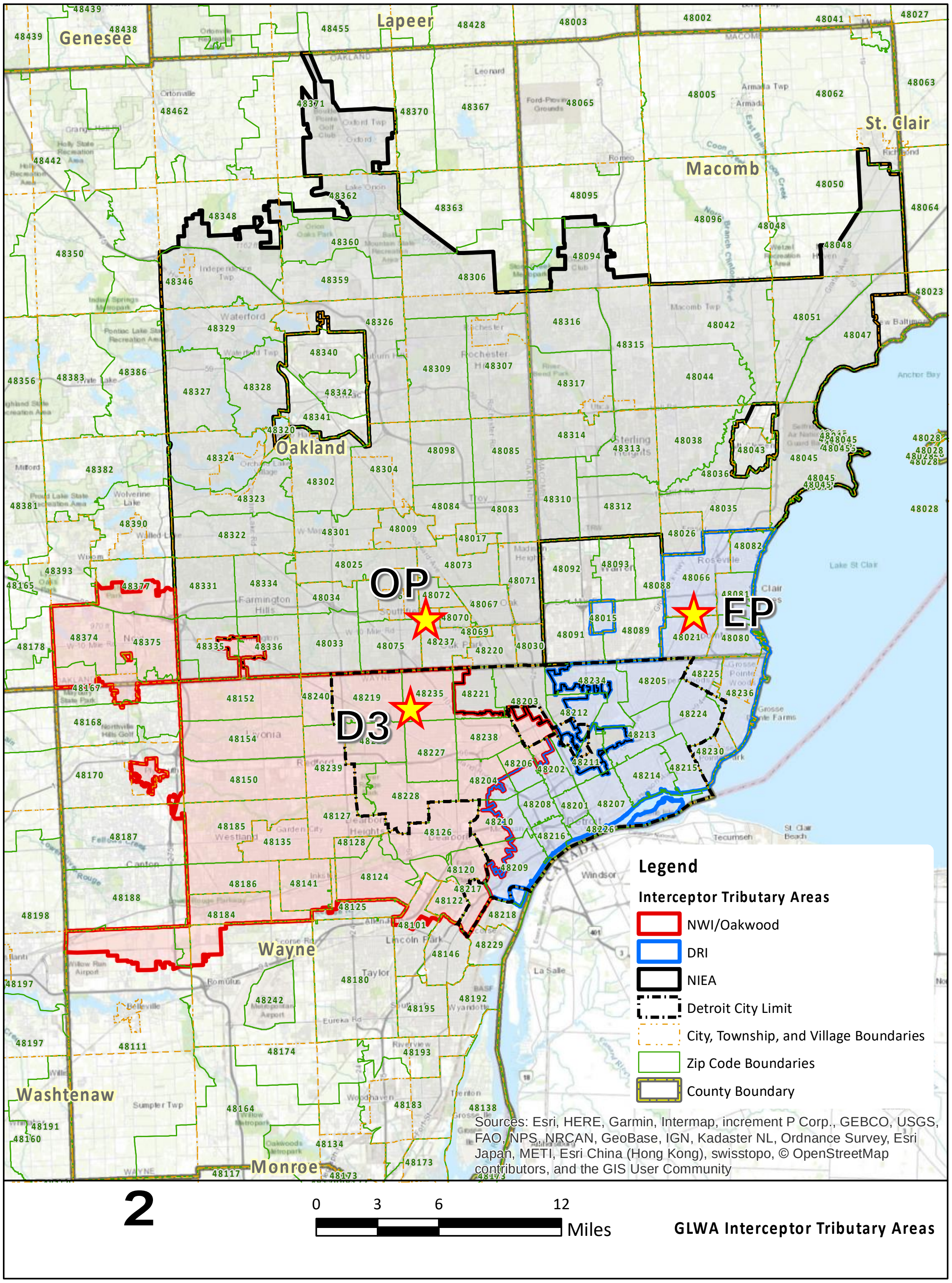

Figure S2. GLWA tributary areas and three selected neighborhood sewersheds

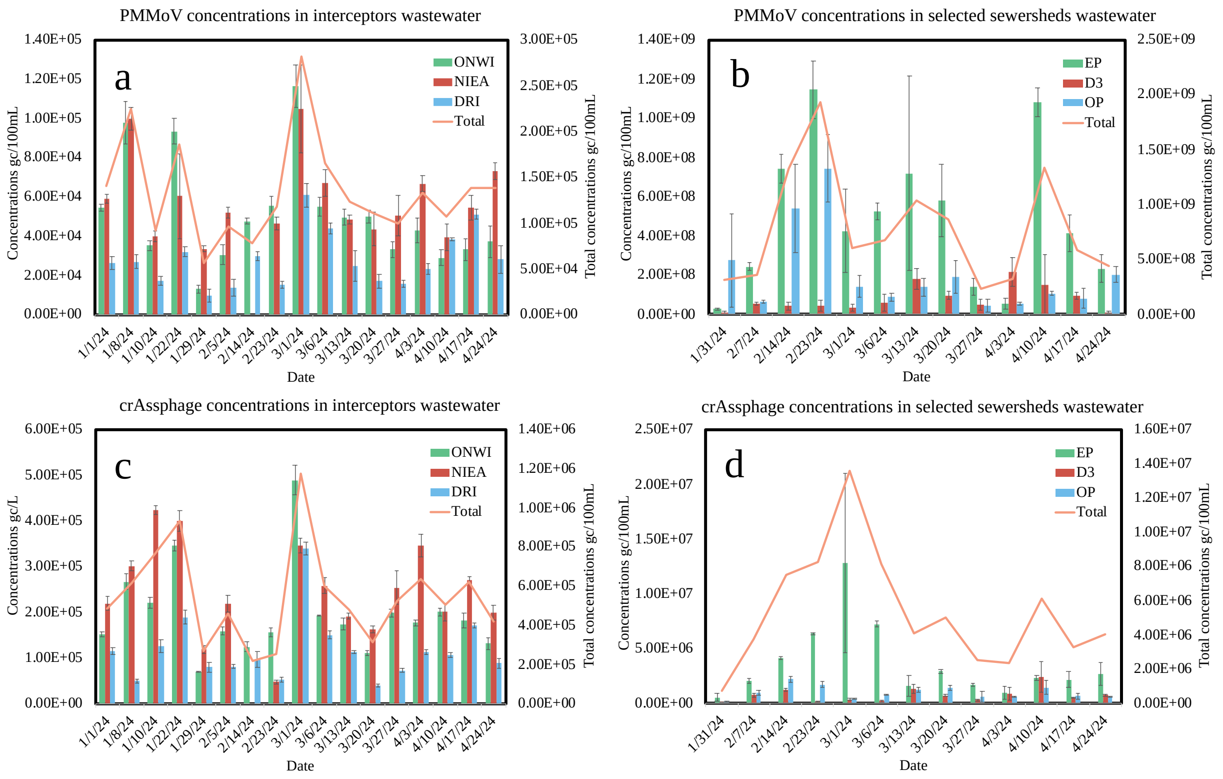

Figure S3. PMMoV and crAssphage concentrations within the study period for interceptors (a, c) and neighborhood selected sewersheds (b, d)

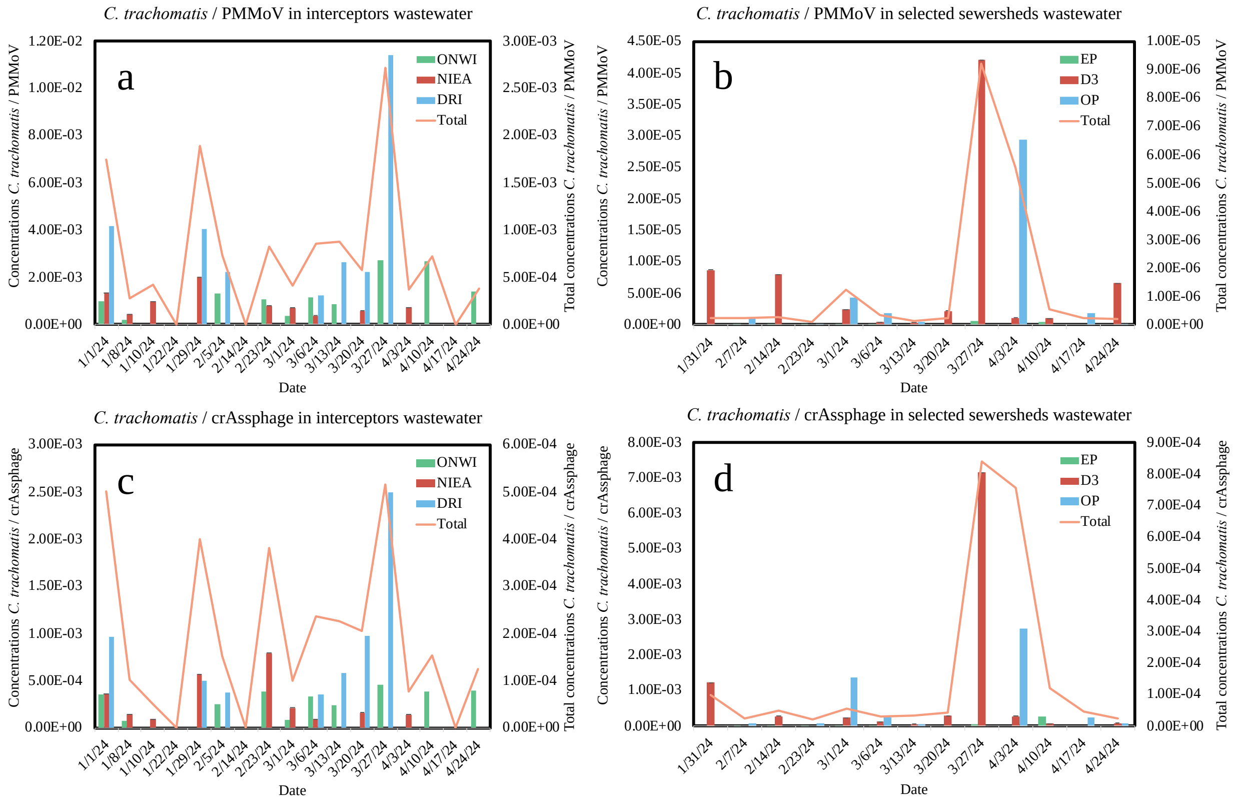

Figure S4. *C. trachomatis* concentrations normalized by PMMoV within the study period for interceptors (a) and neighborhood selected sewersheds (b); *C. trachomatis* concentrations normalized by crAssphage within the study period for interceptors (c) and neighborhood selected sewersheds (d)

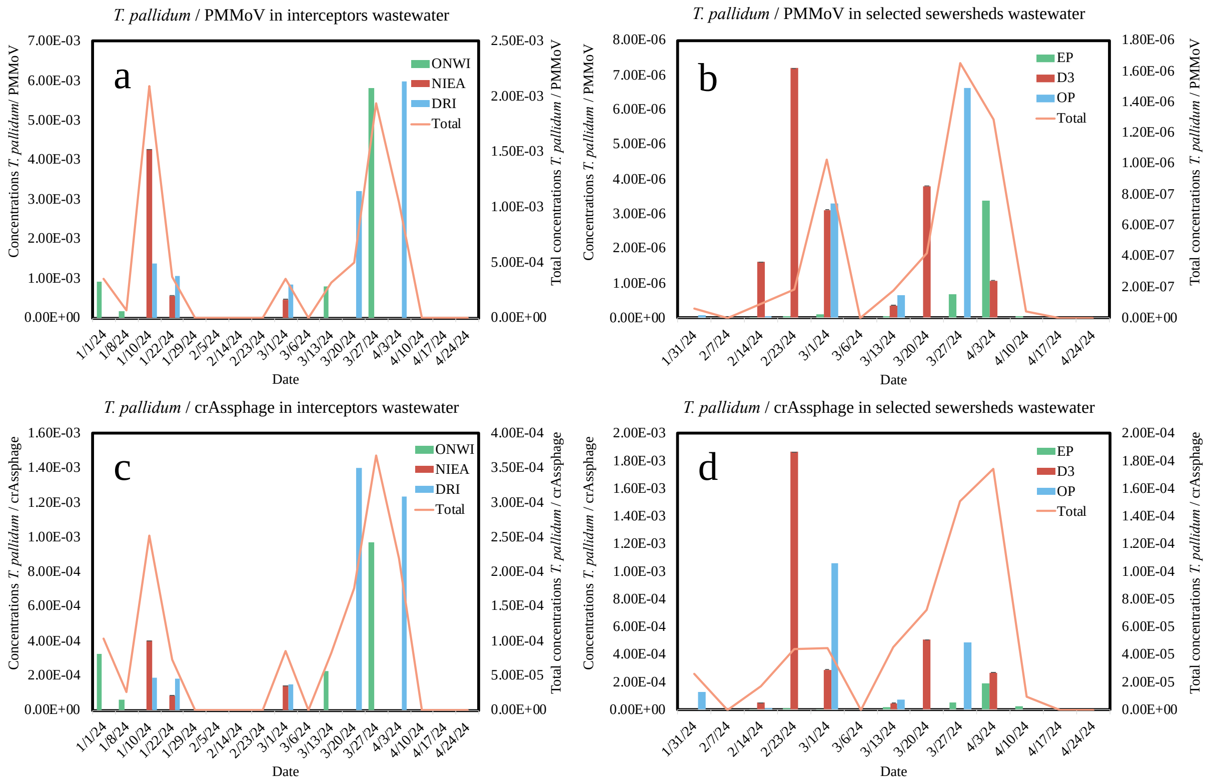

Figure S5. *T. pallidum* concentrations normalized by PMMoV within the study period for interceptors (a) and neighborhood selected sewersheds (b); *T. pallidum* concentrations normalized by crAssphage within the study period for interceptors (c) and neighborhood selected sewersheds (d)

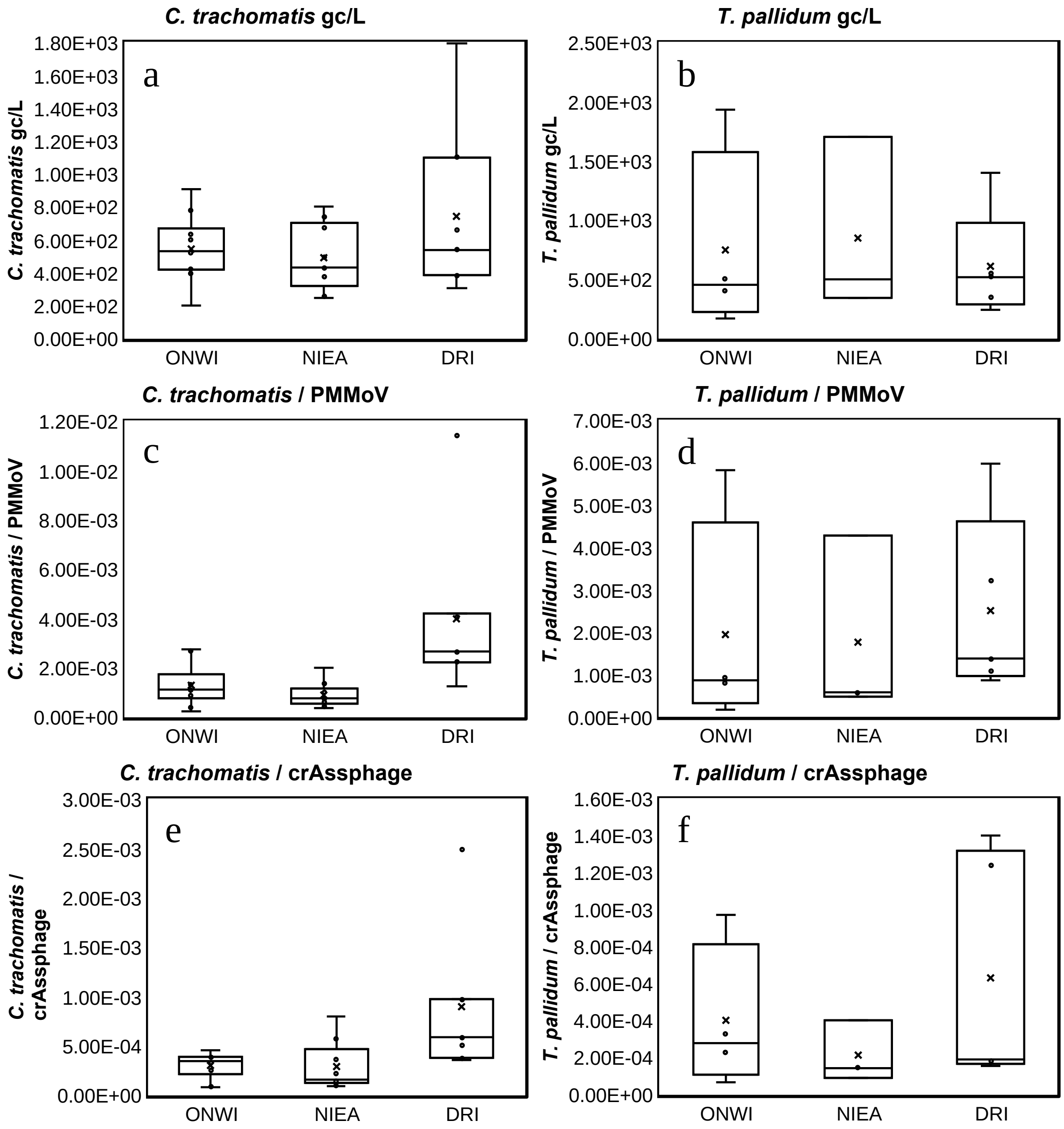

Figure S6. Box plots for *C. trachomatis* and *T. pallidum* concentrations: non-normalized (a, b), normalized by PMMoV (c, d), normalized by crAssphage (e, f) for interceptors

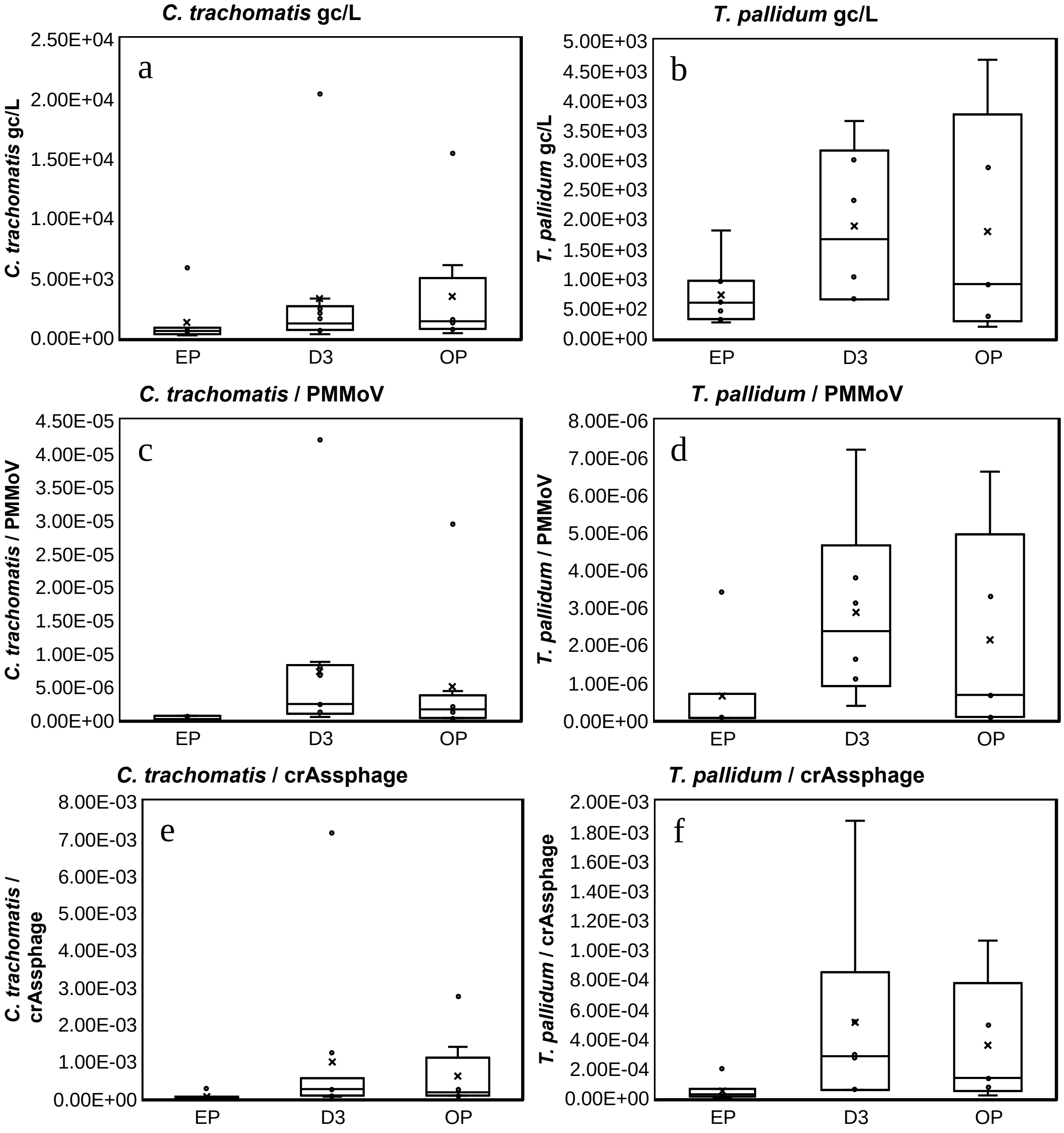

Figure S7. Box plots for *C. trachomatis* and *T. pallidum* concentrations: non-normalized (a, b), normalized by PMMoV (c, d), normalized by crAssphage (e, f) for selected sewersheds

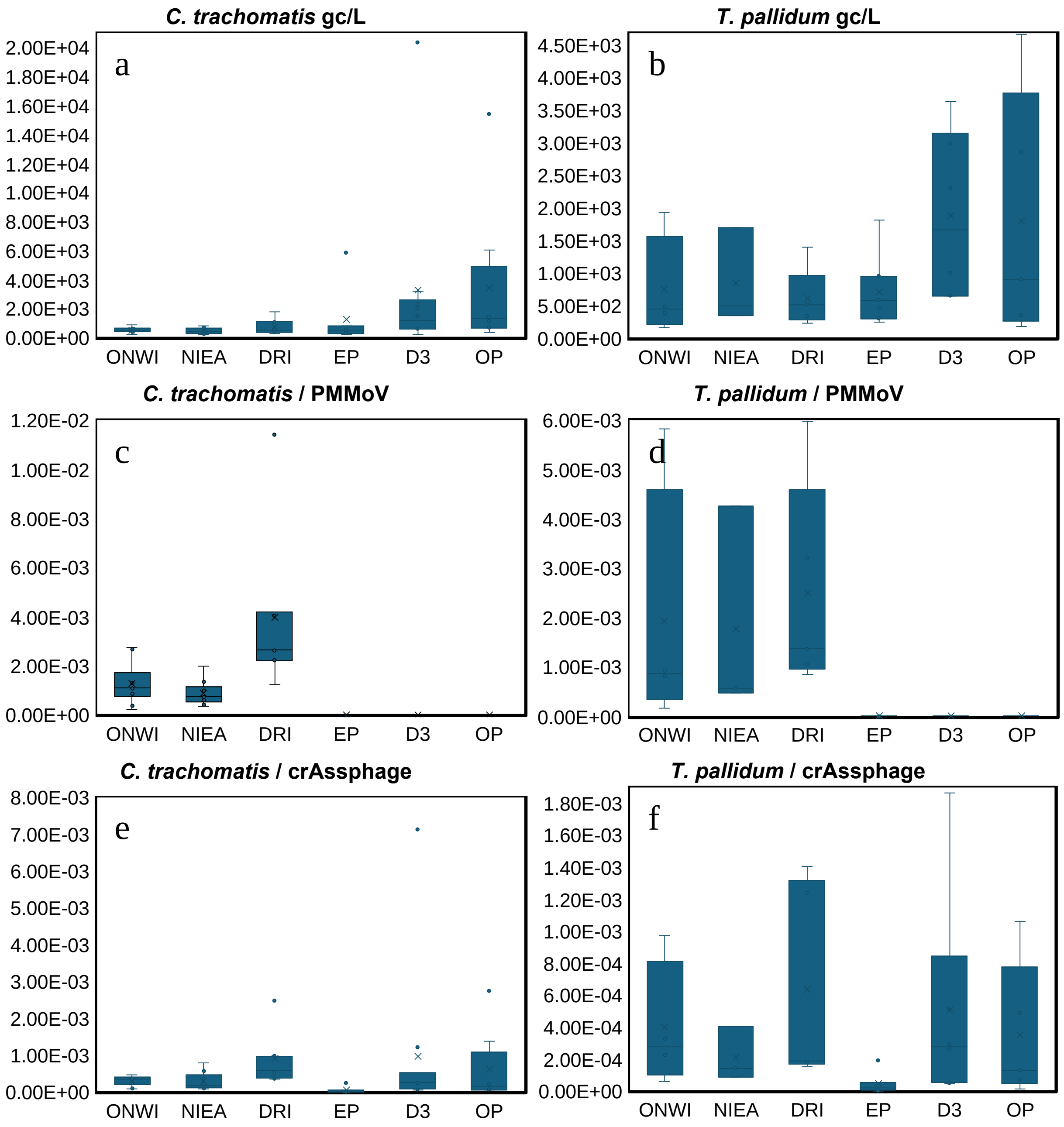

Figure S8. Comparison between interceptors and selected sewersheds for *C. trachomatis* and *T. pallidum* concentrations: non-normalized (a, b), normalized by PMMoV (c, d), normalized by crAssphage (e, f)

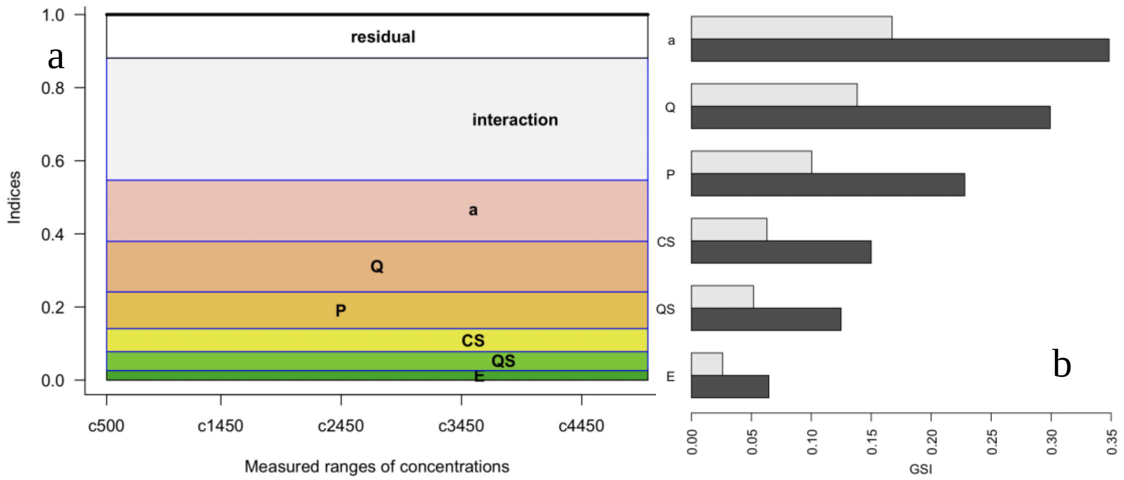

Figure S9. (a) Dynamics of the sensitivity indices of Chlamydia infection estimates with indices normalized to 1. (b) Bar plot of the PCA generalized sensitivity indices of the infection estimates model for Chlamydia (light grey indicates first order indices, dark grey indicates total indices)

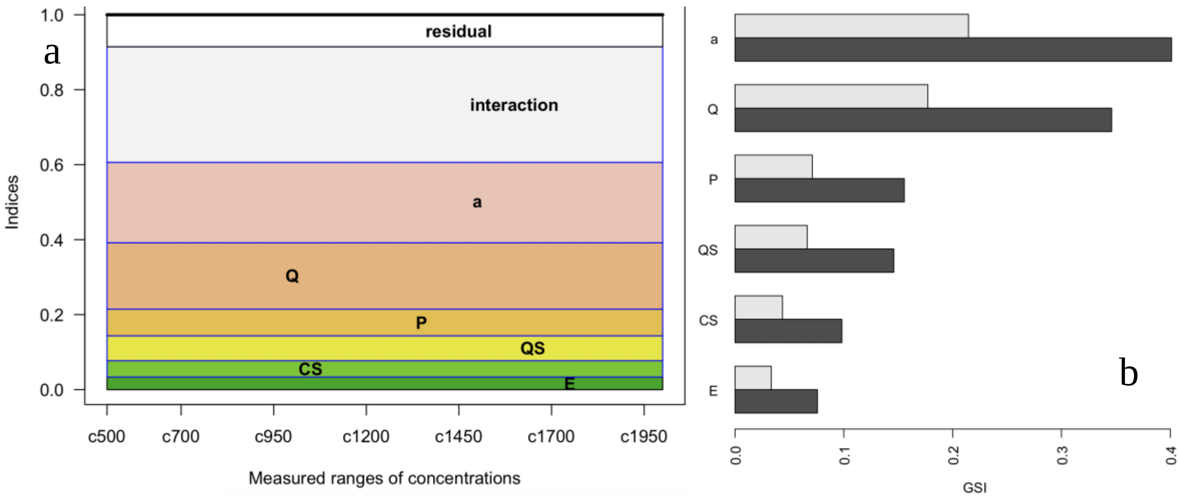

Figure S10. (a) Dynamics of the sensitivity indices of Syphilis infection estimates with indices normalized to 1. (b) Bar plot of the PCA generalized sensitivity indices of the infection estimates model for Syphilis (light grey indicates first order indices, dark grey indicates total indices)

Table S1. Sequences of the primers and probes for targeting *C. trachomatis* and *T. pallidum*

Organism	Target gene	Oligonucleotide Sequence (5’>3’)	References
C. trachomatis	*ompA*	Forward: CATGARTGGCAAGCAAGTTTA	(1,2)
		Reverse: GCAATACCGCAAGATTTTCTAG	
		Probe: FAM-TGTTCACTCCYTACATTGGAGT-BHQ1	
T. pallidum	*polA*	Forward: GGTAGAAGGGAGGGCTAGTA	(3–5)
		Reverse: CTAAGATCTCTATTTTCTATAGGTATGG	
		Probe: FAM-ACACAGCACTCGTCTTCAACTCC-BHQ1	

Table S2. Optimized ddPCR Mastermix reaction for both *C. trachomatis* and *T. pallidum*

Reagents (concentration)	Volume (µL)
Standard solution of Bio-Rad kit	8.8
Forward primer (900 nM)	1.98
Reverse primer (900 nM)	1.98
Probe (250 nM)	1.1
PCR-grade water	2.64
Extracted DNA	5.5

.

Table S3. Optimized ddPCR thermocycling conditions

Name of each step	Conditions for *C. trachomatis*	Conditions for *T. pallidum*
Standard conditions for Bio-Rad kit	25°C for 3 min, 50°C for 60 min, 95°C for 10 min	
Denaturation, annealing, extension	55 Cycles* of 95°C for 10 sec*, 55°C for 20 sec*, 65°C for 40 sec*, and 40°C for 10 sec (Stevens et al., 2010)	50 Cycles* of 95°C for 30 sec*, 55°C for 30 sec*, 72°C for 30 sec* (Koek et al., 2006)
Enzyme deactivation	98°C for 10 min	
Hold	Hold at 4°C for ∞	

Note: *No hot start, 40 µL reaction, and slow ramp speed of 2°C/second are required for all.

Table S4. Centrifugation methods for bacterial concentration from environmental and clinical samples

Bacterial target	Sample matrix	Centrifugation speed and time	References
Chlamydia trachomatis	Endocervical swab sample	≥12,000×*g* for 30 minutes at 4°C	(18)
	Cervical and introital specimens	10,000 rpm for 15 min	(19)
Salmonella Typhi	Influent wastewater grab sample	1-minute 1000xg then supernatant for 15 minutes 4000xg	(20)
Escherichia coli	Surface natural water (river) and wastewater (raw wastewater before treatment)	8000*g*, 10 min, 22 °C	(21)
Legionella pneumophila	Tap water samples	8150 g for 15 min or 3800 g for 30 min	(22)
Enterococcus faecalis	Wastewater samples	12,000×*g* for 1 min	(23)
Leptospira	Water samples	8000 x*g*, 10 min	(24)
Escherichia coli	Cultured medium	8000g, 10 min, 4 °C<br>12,000×g for 10 min	(25)

Table S5. Decay rate constant k (d^{-1}) for different bacteria in the aqueous environment

Bacteria	Method	Environment	Temperature	Decay rate	Reference
E. Coli	RT-qPCR	Seawater	22–24 °C	0.06-1.47	(26)
Enterococci				0.18-0.76	
C. perfringens				0-0.77	
Enterococci (intestinal)	Membrane filtration with incubation on selective media	Seawater	4–20 °C	0.03-1.05	(27)
E. Coli	IDEXX Colilert 18 Quanti-Tray/2000			0.05-1.13	
Campylobacter	Calculations using the Arrhenius equation based on data collected from published studies	Wastewater	20 °C	0.17-0.19	(28)
		Fresh water		1.72-1.88	
		Saline water		1.15-2.75	
Salmonella		Wastewater		0.4-0.52	
		Fresh water		0.37-2.37	
		Saline water		0.74-1.06	
Bacteroidales	Real-time PCR	Seawater	18.3-18.7 °C	0.95-1.11	(29)
		Fresh water		1.37-1.41	
Bifidobacterium adolescentis		Seawater		0.62-0.64	
		Fresh water		0.62-0.7	
E. coli (culturable)	Culture	Seawater		1.24-1.4	
		Fresh water		0.39-0.43	
Enterococci (culturable)		Seawater		0.56-0.88	
		Fresh water		0.67-0.91	
E. coli (culturable)	Culture	Seawater	15.06-19.22 °C	0.5	(30)
Enterococci (culturable)				0.65	
Enterococci	RT-qPCR			0.3	

Table S6. Wastewater in-sewer travel time in GLWA interceptors

Interceptor (hours)	Weighted Average	Minimum	Maximum
DRI	12.3	0.2	41.8
NIEA	22.5	0.7	51.2
ONWI	8.6	0.1	25.9

Table S7. Temperature and pH of wastewater samples collected from the selected sewersheds

Week	Sample site (duplicate samples)	Sample Date	Temperature (ºC)	pH
1/8/24	OP	1/10/24	pH/Temp sensor missing	pH/Temp sensor missing
	OP	1/10/24		
	D3	1/10/24	pH/Temp sensor missing	pH/Temp sensor missing
	D3	1/10/24		
	EP	1/10/24	pH/Temp sensor missing	pH/Temp sensor missing
	EP	1/10/24		
1/22/24	OP	1/24/24	11.1	7.75
	OP	1/24/24		
	D3	1/24/24	12.1	7.41
	D3	1/24/24		
	EP	1/24/24	13.1	7.35
	EP	1/24/24		
1/29/24	OP	1/31/24	12.5	7.75
	OP	1/31/24		
	D3	1/31/24	7.6	7.72
	D3	1/31/24		
	EP	1/31/24	12.3	7.53
	EP	1/31/24		
2/5/24	OP	2/7/24	14.3	7.45
	OP	2/7/24		
	D3	2/7/24	14	7.57
	D3	2/7/24		
	EP	2/7/24	14.3	7.34
	EP	2/7/24		
2/12/24	OP	2/14/24	pH/Temp sensor missing	pH/Temp sensor missing
	OP	2/14/24		
	D3	2/14/24	pH/Temp sensor missing	pH/Temp sensor missing
	D3	2/14/24		
	EP	2/14/24	pH/Temp sensor missing	pH/Temp sensor missing
	EP	2/14/24		
2/19/24	OP	2/21/24	14.9	7.53
	OP	2/21/24		

	D3	2/21/24	15.8	7.62
	D3	2/21/24		
	EP	2/21/24	16.6	7.35
	EP	2/21/24		
2/26/24	OP	2/28/24	12.1	7.78
	OP	2/28/24		
	D3	2/28/24	10.5	7.8
	D3	2/28/24		
	EP	2/28/24	8.7	7.47
	EP	2/28/24		
3/4/24	OP	3/6/24	11.9	7.82
	OP	3/6/24		
	D3	3/6/24	13.1	7.77
	D3	3/6/24		
	EP	3/6/24	11.4	7.64
	EP	3/6/24		
3/11/24	OP	3/13/24	19	7.71
	OP	3/13/24		
	D3	3/13/24	15.8	7.53
	D3	3/13/24		
	EP	3/13/24	21.7	7.6
	EP	3/13/24		
3/18/24	OP	3/20/24	9.5	7.63
	OP	3/20/24		
	D3	3/20/24	9	7.61
	D3	3/20/24		
	EP	3/20/24	11	7.49
	EP	3/20/24		
3/25/24	OP	3/27/24	10.3	7.61
	OP	3/27/24		
	D3	3/27/24	11	7.66
	D3	3/27/24		
	EP	3/27/24	11.3	7.54
	EP	3/27/24		
4/1/24	OP	4/3/24	14.1	7.74

	OP	4/3/24		
	D3	4/3/24	12.9	7.65
	D3	4/3/24		
	EP	4/3/24	15.5	7.63
	EP	4/3/24		
4/8/24	OP	4/10/24	16.8	7.58
	OP	4/10/24		
	D3	4/10/24	17	7.66
	D3	4/10/24		
	EP	4/10/24	18.4	7.48
	EP	4/10/24		
4/16/24	OP	4/18/24	18.3	7.65
	OP	4/18/24		
	D3	4/18/24	16.8	7.52
	D3	4/18/24		
	EP	4/18/24	19.9	7.58
	EP	4/18/24		
4/22/24	OP	4/24/24	13.9	7.64
	OP	4/24/24		
	D3	4/24/24	13.7	7.6
	D3	4/24/24		
	EP	4/24/24	14.7	7.45
	EP	4/24/24		

Table S8. Positive detection of *C. trachomatis* in clinical samples excreted from patients with suspected diseases (* marked values indicate presence rates in urine and are used in formula 2)

Type of samples	Presence (%)	References
Female urine	5.3*	(31)
	6.6*	(32)
	6.0*	(33)
All gender urine	6.2*	(31)
	9.0*	(33)
	9.1*	(32)
Male urine	13.4*	(32)
	14.4*	(33)
	7.3*	(31)
Male first voided urine specimens	8.9*	(19)
Female urine combined with vaginal swab	8.3	(19)
All gender genital ulcer swabs	10.5	(34)
Female cervical and introital specimens	12.3	(19)
Female vaginal swabs	12.2	(35)
	18.4	(36)

Table S9. Positive detection of *T. pallidum* in clinical samples excreted from patients with suspected diseases (* marked values indicate presence rates in urine and are used in formula 2)

Type of samples	Presence (%)	References
Male lesion swabs	0.3	(37)
All gender genital ulcer swabs	6.7	(34)
Male urine (early latent syphilis)	12.8*	(5)
All gender ano-rectal swabs (primary syphilis)	13.0	(3)
All gender genital ulcer swabs	13.4	(4)
Male ano-rectal swabs (secondary syphilis)	18.6	(5)
Male genital, anal or oral ulcers	20.8	(38)
Male ano-rectal swabs (early latent syphilis)	24.4	(5)
All ano-rectal swabs (secondary syphilis)	25.6	(3)
Male urine (secondary syphilis)	37.1*	(5)
Male genital, anal or oropharyngeal ulcers	47.0	(39)

Table S10. Incubation time of *C. trachomatis*

Incubation time	References
5-10 days	(40)
7-21 days	lacounty.gov, ndhealth.gov, iowa.gov, epi.utah.gov, odh.ohio.gov
7-14 days	vic.gov.au, nsw.gov.au, gov.mb.ca
mean of 21 days	mass.gov
7-28 days	(41)
2-60 days	ashm.org.au
1-3 weeks	oregon.gov
1-5 weeks	nj.gov
14-21 days, maximum 6 weeks	healthunit.org, gnb.ca
7-14 days for trachoma and genital infections, 3-30 days for LGV	Public Health Agency of Canada (canada.ca)

Table S11. Demographic characteristics of the neighborhood sewersheds in the DMA, MI, USA

Sites	County	City or township	Zip	Area	Population	Density	Black	Hispanic	White	Poverty	Median age	Total household income
EP	Macomb	Eastpointe	48021	278	2400	9	37%	5%	54%	5%	40	56450
D3	Wayne	Detroit	48235	127	1300	10	95%	0%	2%	44%	32	22100
OP	Oakland	Oak Park	48237	286	2270	8	85%	3%	6%	15%	42	51680

Notes: units for Area, Density, and Total household income are acres, people per acre, and USD, respectively.

Table S12. dMIQE2020 checklist

ITEM TO CHECK	PROVIDED	COMMENT
	**Y/N**	
**1. SPECIMEN**		
Detailed description of specimen type and numbers	**Y**	Section 2.3 and Table S7
Sampling procedure (including time to storage)	**Y**	Section 2.3
Sample aliquotation, storage conditions and duration	**Y**	Section 2.3
**2. NUCLEIC ACID EXTRACTION**		
Description of extraction method including amount of sample processed	**Y**	Section 2.4
Volume of solvent used to elute/resuspend extract	**Y**	Section 2.4
Number of extraction replicates	**Y**	Section 2.4
Extraction blanks included?	**N**	N/A
**3. NUCLEIC ACID ASSESSMENT AND STORAGE**		
Method to evaluate quality of nucleic acids	**N**	N/A
Method to evaluate quantity of nucleic acids (including molecular weight and calculations when using mass)	**N**	N/A
Storage conditions: temperature, concentration, duration, buffer, aliquots	**Y**	Section 2 Materials and Methods
Clear description of dilution steps used to prepare working DNA solution	**Y**	Section 2.1
**4. NUCLEIC ACID MODIFICATION**	**NA**	N/A
Template modification (digestion, sonication, pre-amplification, bisulphite etc.)	**N**	N/A
Details of repurification following modification if performed	**N**	N/A
**5. REVERSE TRANSCRIPTION**	**NA**	N/A
cDNA priming method and concentration	**N**	N/A
One or two step protocol (include reaction details for two step)	**N**	N/A
Amount of RNA added per reaction	**N**	N/A

Detailed reaction components and conditions	**N**	N/A
Estimated copies measured with and without addition of RT*	**N**	N/A
Manufacturer of reagents used with catalogue and lot numbers	**N**	N/A
Storage of cDNA: temperature, concentration, duration, buffer and aliquots	**N**	N/A
**6. dPCR OLIGONUCLEOTIDES DESIGN AND TARGET INFORMATION**		
Sequence accession number or official gene symbol	**Y**	Section 2.1
Method (software) used for design and *in silico* verification	**N**	N/A
Location of amplicon	**Y**	Section 2.1 and provided citations
Amplicon length	**Y**	Section 2.1 and provided citations
Primer and probe sequences (or amplicon context sequence)**	**Y**	Supporting Information
Location and identity of any modifications	**N**	N/A
Manufacturer of oligonucleotides	**Y**	Section 2.1
**7. dPCR PROTOCOL**		
Manufacturer of dPCR instrument and instrument model	**Y**	Section 2.5
Buffer/kit manufacturer with catalogue and lot number	**Y**	Section 2.5
Primer and probe concentration	**Y**	Section 2.5
Pre-reaction volume and composition (incl. amount of template and if restriction enzyme added)	**Y**	Section 2.5
Template treatment (initial heating or chemical denaturation)	**Y**	Section 2.5
Polymerase identity and concentration, Mg++ and dNTP concentrations***	**N**	N/A
Complete thermocycling parameters	**Y**	Section 2.5
**8. ASSAY VALIDATION**		
Details of optimisation performed	**Y**	Section 2 Materials and Methods
Analytical specificity (vs. related sequences) and limit of blank (LOB)	**Y**	Section 2.6
Analytical sensitivity/LoD and how this was evaluated	**Y**	Section 2.6
Testing for inhibitors (from biological matrix/extraction)	**N**	Dilution tests were profermed but

		details were not included
**9. DATA ANALYSIS**		
Description of dPCR experimental design	**Y**	Section 2 Materials and Methods
Comprehensive details negative and positive of controls (whether applied for QC or for estimation of error)	**Y**	Section 2 Materials and Methods
Partition classification method (thresholding)	**N**	N/A
Examples of positive and negative experimental results (including fluorescence plots in supplemental material)	**N**	N/A
Description of technical replication	**Y**	Section 2 Materials and Methods
Repeatability (intra-experiment variation)	**Y**	Section 2 Materials and Methods
Reproducibility (inter-experiment/user/lab etc. variation)	**Y**	Section 2 Materials and Methods
Number of partitions measured (average and standard deviation)	**N**	N/A
Partition volume	**N**	N/A
Copies per partition (λ or equivalent) (average and standard deviation)	**Y**	Section 2.7
dPCR analysis program (source, version)	**Y**	Section 2.5
Description of normalisation method	**Y**	Section 2.7
Statistical methods used for analysis	**Y**	Section 2.7
Data transparency	raw data available on request:	Figure 1 and raw data available on request at xagorara@msu.edu

References:

1. Chin Quee JE. Using Wastewater-Based Epidemiology to Study Chlamydia Occurrence on a College Campus. 2023;

2. Stevens MP, Twin J, Fairley CK, Donovan B, Tan SE, Yu J, et al. Development and Evaluation of an *ompA* Quantitative Real-Time PCR Assay for *Chlamydia trachomatis* Serovar Determination. J Clin Microbiol. 2010 Jun;48(6):2060–5.

3. Heymans R, van der Helm JJ, de Vries HJC, Fennema HSA, Coutinho RA, Bruisten SM. Clinical Value of Treponema pallidum Real-Time PCR for Diagnosis of Syphilis. J Clin Microbiol. 2010 Feb;48(2):497–502.

4. Koek AG, Bruisten SM, Dierdorp M, van Dam AP, Templeton K. Specific and sensitive diagnosis of syphilis using a real-time PCR for Treponema pallidum. Clinical Microbiology and Infection. 2006 Dec;12(12):1233–6.

5. Nieuwenburg SA, Zondag HCA, Bruisten SM, Jongen VW, Schim van der Loeff MF, van Dam AP, et al. Detection of Treponema pallidum DNA During Early Syphilis Stages in Peripheral Blood, Oropharynx, Ano-Rectum and Urine as a Proxy for Transmissibility. Clinical Infectious Diseases. 2022 Sep 29;75(6):1054–62.

6. Salle R, Mayslich C, Grange PA, Leducq V, Ollagnier G, Heller U, et al. Specific detection of Treponema pallidum in clinical samples: validation of a qPCR assay combining two genomic targets. Sex Transm Infect [Internet]. 2023 Mar 1;99(2):91. Available from: http://sti.bmj.com/content/99/2/91.abstract

7. Pierson-Perry JF, Vaks JE, Vore TEK, Durham AP, Fischer C, Gutenbrunner C, et al. Evaluation of detection capability for clinical laboratory measurement procedures; approved guideline. Clinical Laboratory Standards Institute; 2012.

8. Lamboni M, Monod H, Makowski D. Multivariate sensitivity analysis to measure global contribution of input factors in dynamic models. Reliab Eng Syst Saf. 2011 Apr;96(4):450–9.

9. Kitajima M, Sassi HP, Torrey JR. Pepper mild mottle virus as a water quality indicator. NPJ Clean Water. 2018 Oct 15;1(1):19.

10. Greenwald HD, Kennedy LC, Hinkle A, Whitney ON, Fan VB, Crits-Christoph A, et al. Tools for interpretation of wastewater SARS-CoV-2 temporal and spatial trends demonstrated with data collected in the San Francisco Bay Area. Water Res X. 2021 Aug;12:100111.

11. Rao G, Capone D, Zhu K, Knoble A, Linden Y, Clark R, et al. Simultaneous detection and quantification of multiple pathogen targets in wastewater. PLOS Water. 2024 Feb 1;3(2):e0000224.

12. Holm RH, Nagarkar M, Yeager RA, Talley D, Chaney AC, Rai JP, et al. Surveillance of RNase P, PMMoV, and CrAssphage in wastewater as indicators of human fecal concentration across urban sewer neighborhoods, Kentucky. FEMS Microbes. 2022 Mar 31;3.

13. Boehm AB, Wolfe MK, White B, Hughes B, Duong D, Banaei N, et al. Human norovirus (HuNoV) GII RNA in wastewater solids at 145 United States wastewater treatment plants: Comparison to positivity rates of clinical specimens and modeled estimates of HuNoV GII shedders. medRxiv [Internet]. 2023 Jan 1;2023.05.02.23289421. Available from: http://medrxiv.org/content/early/2023/05/05/2023.05.02.23289421.abstract

14. Nagarkar M, Keely SP, Jahne M, Wheaton E, Hart C, Smith B, et al. SARS-CoV-2 monitoring at three sewersheds of different scales and complexity demonstrates distinctive relationships between wastewater measurements and COVID-19 case data. Science of The Total Environment. 2022 Apr;816:151534.

15. Zhao L, Zou Y, Li Y, Miyani B, Spooner M, Gentry Z, et al. Five-week warning of COVID-19 peaks prior to the Omicron surge in Detroit, Michigan using wastewater surveillance. Science of The Total Environment. 2022 Jun;157040.

16. Zhao L, Guzman HP, Xagoraraki I. Comparative analyses of SARS-CoV-2 RNA concentrations in Detroit wastewater quantified with CDC N1, N2, and SC2 assays reveal optimal target for predicting COVID-19 cases. Science of The Total Environment [Internet]. 2024;945:174140. Available from: https://www.sciencedirect.com/science/article/pii/S0048969724042888

17. US EPA. (2014). Method 1603: Escherichia coli (E. coli) in Water by Membrane Filtration Using Modified membrane-Thermotolerant Escherichia coli Agar (Modified mTEC) [Internet]. [cited 2023 Oct 7]. Available from: https://www.epa.gov/sites/default/files/2015-08/documents/method_1603_2009.pdf

18. Somboonna N, Choopara I, Arunrut N, Sukhonpan K, Sayasathid J, Dean D, et al. Rapid and sensitive detection of Chlamydia trachomatis sexually transmitted infections in resource-constrained settings in Thailand at the point-of-care. PLoS Negl Trop Dis. 2018 Dec 20;12(12):e0006900.

19. Mania-Pramanik J, Potdar S, Kerkar S. Diagnosis of Chlamydia trachomatis infection. J Clin Lab Anal. 2006 Jan 8;20(1):8–14.

20. Zhou N, Ong A, Fagnant-Sperati C, Harrison J, Kossik A, Beck N, et al. Evaluation of Sampling and Concentration Methods for Salmonella enterica Serovar Typhi Detection from Wastewater. Am J Trop Med Hyg. 2023 Mar 1;108(3):482–91.

21. El Boujnouni H, Nait Balla K, Belkadi B, Rahouti M. Comparison between the recovery rate of three concentration protocols of water samples intended for analysis by Molecular

Biology: Membrane filtration, filtration on gauze pad and centrifugation. Saudi J Biol Sci. 2022 Mar;29(3):1592–7.

22. Villari P, Motti E, Farullo C, Torre I. Comparison of conventional culture and PCR methods for the detection of Legionella pneumophila in water. Lett Appl Microbiol. 1998 Aug;27(2):106–10.

23. Varma M, Field R, Stinson M, Rukovets B, Wymer L, Haugland R. Quantitative real-time PCR analysis of total and propidium monoazide-resistant fecal indicator bacteria in wastewater. Water Res. 2009 Nov;43(19):4790–801.

24. Lall C, Kumar KV, Raj RV, Vedhagiri K, Vijayachari P. Prevalence and Diversity of Leptospires in Different Ecological Niches of Urban and Rural Areas of South Andaman Island. Microbes Environ. 2016;31(1):79–82.

25. Fu Y, Ye Z, Jia Y, Fan J, Hashmi MZ, Shen C. An Optimized Method to Assess Viable Escherichia coli O157:H7 in Agricultural Soil Using Combined Propidium Monoazide Staining and Quantitative PCR. Front Microbiol. 2020 Jul 31;11.

26. Zhang Q, He X, Yan T. Differential Decay of Wastewater Bacteria and Change of Microbial Communities in Beach Sand and Seawater Microcosms. Environ Sci Technol. 2015 Jul 21;49(14):8531–40.

27. Eregno FE, Tryland I, Myrmel M, Wennberg A, Oliinyk A, Khatri M, et al. Decay rate of virus and faecal indicator bacteria (FIB) in seawater and the concentration of FIBs in different wastewater systems. Microb Risk Anal. 2018 Apr;8:14–21.

28. Guo Y, Sivakumar M, Jiang G. Decay of four enteric pathogens and implications to wastewater-based epidemiology: Effects of temperature and wastewater dilutions. Science of The Total Environment. 2022 May;819:152000.

29. Jeanneau L, Solecki O, Wéry N, Jardé E, Gourmelon M, Communal PY, et al. Relative Decay of Fecal Indicator Bacteria and Human-Associated Markers: A Microcosm Study Simulating Wastewater Input into Seawater and Freshwater. Environ Sci Technol. 2012 Feb 21;46(4):2375–82.

30. Mattioli MC, Sassoubre LM, Russell TL, Boehm AB. Decay of sewage-sourced microbial source tracking markers and fecal indicator bacteria in marine waters. Water Res. 2017 Jan;108:106–14.

31. Božičević I, Grgić I, Židovec-Lepej S, Čakalo JI, Belak-Kovačević S, Štulhofer A, et al. Urine-based testing for Chlamydia trachomatis among young adults in a population-based survey in Croatia: Feasibility and prevalence. BMC Public Health. 2011 Dec 14;11(1):230.

32. Møller JK, Pedersen LN, Persson K. Comparison of the Abbott RealTi *m* e CT New Formulation Assay with Two Other Commercial Assays for Detection of Wild-Type and New Variant Strains of *Chlamydia trachomatis*. J Clin Microbiol. 2010 Feb;48(2):440–3.

33. Møller JK, Pedersen LN, Persson K. Comparison of Gen-Probe Transcription-Mediated Amplification, Abbott PCR, and Roche PCR Assays for Detection of Wild-Type and Mutant Plasmid Strains of *Chlamydia trachomatis* in Sweden. J Clin Microbiol. 2008 Dec;46(12):3892–5.

34. Tshaka TR, Singh R, Apalata TR, Mbulawa ZZA. Aetiology of genital ulcer disease and associated factors among Mthatha public clinic attendees. S Afr J Infect Dis. 2022 Dec 7;37(1).

35. Ngobese B, Swe-Han KS, Tinarwo P, Abbai NS. Significant Associations between Chlamydia trachomatis and Neisseria gonorrhoeae Infections in Human Immunodeficiency Virus-Infected Pregnant Women. Infect Dis Obstet Gynecol. 2022 Jun 17;2022:1–13.

36. Pickett ML, Visotcky A, Brazauskas R, Ledeboer NA, Drendel AL. Can a Clean Catch Urine Sample Be Used to Diagnose Chlamydia and Gonorrhea in Adolescent Females? Journal of Adolescent Health. 2021 Oct;69(4):574–8.

37. Dubourg G, Edouard S, Prudent E, Fournier PE, Raoult D. Incidental Syphilis Diagnosed by Real-Time PCR Screening of Urine Samples. J Clin Microbiol. 2015 Nov;53(11):3707–8.

38. Shields M, Guy RJ, Jeoffreys NJ, Finlayson RJ, Donovan B. A longitudinal evaluation of Treponema pallidumPCR testing in early syphilis. BMC Infect Dis. 2012 Dec 17;12(1):353.

39. Glatz M, Juricevic N, Altwegg M, Bruisten S, Komericki P, Lautenschlager S, et al. A multicenter prospective trial to asses a new real-time polymerase chain reaction for detection of Treponema pallidum, herpes simplex-1/2 and Haemophilus ducreyi in genital, anal and oropharyngeal ulcers. Clinical Microbiology and Infection. 2014 Dec;20(12):O1020–7.

40. O’Connell CM, Ferone ME. Chlamydia trachomatis Genital Infections. Microbial Cell. 2016 Sep 5;3(9):390–403.

41. Jones RE, Lopez KH. Sexually Transmitted Diseases. In: Human Reproductive Biology. Elsevier; 2014. p. 323–47.

42. Gendron L, Verreault D, Veillette M, Moineau S, Duchaine C. 2010. Evaluation of filters for the sampling and quantification of RNA phage aerosols. Aerosol Sci. Technol. 44:893–901.
